# Prevention of Infections in Cardiac Surgery (PICS)-Prevena Study – A pilot/vanguard factorial cluster cross-over RCT

**DOI:** 10.1371/journal.pone.0338300

**Published:** 2025-12-15

**Authors:** Thomas C. Scheier, Richard Whitlock, Mark Loeb, Philip James Devereaux, Andre Lamy, Michael McGillion, MacKenzie Quantz, Ingrid Copland, Shun-Fu Lee, Dominik Mertz

**Affiliations:** 1 Population Health Research Institute, McMaster University and Hamilton Health Sciences, Hamilton, ON, Canada; 2 Department of Health Research Methodology, Evidence, and Impact, Faculty of Health Sciences, McMaster University, Hamilton, ON, Canada; 3 Division of Infectious Diseases, Department of Medicine, McMaster University, Hamilton, ON, Canada; 4 World Health Research Trust, Hamilton, ON, Canada; 5 Department of Surgery, McMaster University, Hamilton, ON, Canada; 6 Department of Surgery, Division of Cardiac Surgery, Schulich School for Medicine & Dentistry, London, ON, Canada; Tel Aviv University School of Medicine, ISRAEL

## Abstract

Sternal surgical site infections after cardiac surgery can lead to significant morbidity, mortality, and cost. The effects of negative pressure wound management and adding vancomycin as perioperative antimicrobial prophylaxis are unknown. The PICS-PREVENA pilot/vanguard trial, a 2x2 factorial, open label, cluster-randomized crossover trial with 4 periods, was conducted at two major cardiac surgery hospitals in Ontario, Canada. Sites were randomized to one of eight sequences of the four study arms (Cefazolin or Cefazolin + Vancomycin (not analyzed) and standard wound dressing or a negative pressure 3M Prevena incision management system (Prevena). Only diabetic or obese patients were eligible for the latter comparison. This trial investigated feasability including adherence to protocol of each intervention (goal: > 90% each) and loss to follow-up (goal: < 10%). Among the 4107 included patients, 2230 were obese/diabetic (1208 standard wound dressing period, 1022 during Prevena period). Compliance to wound management and antimicrobial prophylaxis was 68.1% and 98.7%, respectively. Loss to follow-up was 3.6%. Deep/organ-space sternal surgical site infections occurred in 16 (1.6%) patients in the Prevena allocated periods and in 17 (1.4%) patients in the standard wound dressing allocated periods (OR= 1.11, 95% CI: 0.56–2.20). Other clinical outcomes did not suggest a difference and a post-hoc as-treated analysis showed similar results. This study showed challenges with introducing a novel technology as standard of care, with non-compliance mostly driven by one of the sites. No firm conclusions should be drawn regarding the effectiveness of Prevena, as this vanguard trial was not powered for clinical outcomes.

## Introduction

The prevalence of surgical site infections (SSI), one of the most common infections, varies between 0.6–9.5%, depending on the surgical procedure [1]. The occurrence of SSIs is associated with long-term harm, increased mortality, and cost [2–4]. In cardiac surgery, SSIs include local infections at the thoracic and sternal wounds (s-SSI) and infections at vein harvesting sites, which both are reported in about 2% of patients [5,6]. Obese patients or patients with diabetes mellitus are at increased risk for s-SSI after cardiac surgery [6–8].

The multiple interventions, which are recommended to prevent SSIs, can be divided into preoperative, intraoperative, and postoperative interventions [9]. The use of incisional negative pressure wound dressing therapy, a postoperative measure, was found to be associated with a probable decrease in SSI compared to standard dressing in a Cochrane review of randomized controlled trials [10]. However, data regarding cardiac surgery, either for the sternal or vein harvest surgical site, is limited and the low quality of existing studies highlights the need for large, randomised trials [10,11]. The combination of cluster randomization, factorial- and crossover-designs can make large trials more feasible and efficient, provide data in real-world settings, investigate multiple interventions, and control cluster level confounding variables by having each cluster act as its own control [12–14].

The *Prevention of Infections in Cardiac Surgery (PICS)-PREVENA Pilot/Vanguard study* is a multicenter cluster-randomized 2x2 factorial crossover trial investigating antimicrobial prophylaxis (cefazolin and vancomycin prophylaxis vs cefazolin prophylaxis) and the impact of the negative pressure wound treatment (negative pressure Prevena dressing vs standard wound dressing) on the occurrence of SSIs in patients having cardiac surgery. Here we report the feasibility outcomes of adherence to protocol and loss to follow-up as well as the clinical endpoints comparing Prevena to standard wound dressing (S1 Appendix).

## Materials and methods

Reporting of this trial followed the CONSORT checklist – extension for pilot and feasibility trials [15]. (S2 Appendix).

### Trial design and oversight

PICS-Prevena is a 2x2 factorial, open label, cluster-randomized crossover trial (Clinicaltrials.gov, NCT03402945) conducted at two major cardiac surgery hospitals in Ontario, Canada. Hospitals performing more than 300 cardiac surgeries a year were eligible. Clinical Trial Ontario (CTO) approved the study and waived the need for individual patient consent. The study design was adapted from the Prevention of Infections in Cardiac Surgery study, which is still underway [13] (Clinicaltrials.gov, NCT02285140).

The trial was funded by KCI Inc. USA. The industry sponsor had no role in conducting the trial other than supporting education on their product at the study site. The industry sponsor did provide input on the study design, but all final decisions were made by the academic investigators. The industry sponsor had no role in data collection, analysis and had no access to the raw data. The industry sponsor had the right to review and provide feedback on the manuscript before submission, but all final decisions were made by the academic investigators.

The target sample size for the pilot/vanguard phase was 4,000 patients, with roughly 500 patients per each of the four treatment allocations within a 2x2 factorial design at each of the two participating sites resulting in 95% confidence intervals (CIs) for the feasibility outcomes within a small margin of less than ± 1%. Such narrow confidence intervals for feasibility outcomes would not necessarily be needed. However, given that randomization was conducted at the cluster level, we needed to enroll at least two sites to gain the necessary insight into potential challenges with the feasibility of the study design for a potential full trial.

### Eligibility

All patients ≥18 years undergoing open-heart surgery with sternotomy, including minimally invasive sternotomies, during the study period were included. Patients were excluded if they were on systemic antibiotics or if they had an active bacterial infection at the time of surgery, were previously enrolled in this trial, were known to be colonized with MRSA, had a beta-lactam or vancomycin allergy precluding the use of cefazolin or vancomycin, or were participating in other studies that may interfere with this trial. Furthermore, patients with sensitivity to silver received a standard wound dressing in all study arms as Prevena is contraindicated in these patients.

### Randomization

The hospitals were randomly assigned using computer-based randomization to one of eight potential sequences of the four treatment allocations. These four were: i) cefazolin prophylaxis plus Prevena, ii) cefazolin and vancomycin prophylaxis plus Prevena, iii) cefazolin prophylaxis plus standard wound dressing, and iv) cefazolin and vancomycin prophylaxis plus standard wound dressing (S1 Fig). The wound management and antibiotic prophylaxis strategy became standard of care for patients undergoing cardiac surgery during a respective study period. Based on the increased risk for s-SSI, the subgroup of patients who were obese (BMI>=30 kg/m^2^) or diabetic were eligible for Prevena, and as such the comparison of the wound dressing strategy is limited to this population.

The hospital was informed about the next treatment allocation four weeks before the implementation. Before a new study arm was introduced, there was a wash-in phase of four weeks for the new antibiotic prophylaxis. For treatment allocations where Prevena was required, the use of Prevena was introduced in the last week of the wash-in period. The wash-in periods should allow for full-implementation of the interventions as new standard of care and participants undergoing surgery during this time will not be included. To guarantee patient safety, surgeons were allowed to deviate from the standard of care as determined by the treatment allocation whenever deemed necessary.

### Interventions

#### Antibiotic prophylaxis.

Cefazolin was administered at a dose of 2g (3g if body weight >120 kg) within an hour of the surgery, four hours later or at wound closure (whatever came first), followed by two post-operative dose every 8 hours. Vancomycin was given at a dose of roughly 15 mg/kg and repeated once at 12 hours after the first dose.

#### Wound management.

Prevena was applied to sternal sites and, in case of open saphenous vein harvest, the harvest sites of eligible patients at the end of the procedure. The device was used, as recommended by the manufacturer, for 7 days with a continuous negative pressure at −125 mmHg. User manuals were provided to the participants. Standard wound dressing referred to non-negative pressure wound dressing per standard of care at the hospital.

### Data collection

Data was collected from health records and captured in an online research platform. To complete the 90-day follow-up after surgery, data was obtained through a phone call if no follow-up visit was scheduled. Surgical site infections were defined according to the criteria of the Centers for Disease Control and Prevention/ National Healthcare Safety Network definitions [16].

### Outcomes

The outcomes to assess feasibility of this trial design were loss to follow-up (goal: < 10%), adherence to the wound management system (goal: > 90%), and antibiotic regimen as per protocol (goal: > 90%). The primary clinical outcome of the Prevena vs. standard wound dressing arm was a composite of deep incisional or organ/space s-SSI within 90-days. Key secondary clinical effectiveness outcomes were all s-SSI, SSI at the leg site (if open venous saphenous harvest), wound dehiscence, pain intensity at day 5, length of ICU and hospital stay, acute kidney injury and all-cause mortality and mortality in patients with active infection within 90 days. In addition, serious adverse device effects (SADEs) were collected during Prevena periods. Detailed information is provided in the statistical analysis plan (S3 Appendix).

A blinded outcome adjudication committee reviewed all cases with potential signs or symptoms of an infection that could not be clearly attributed to an infectious focus other than the surgical site(s), and all patients with (suspected) s-SSI.

We remain blinded to the results of the two antibiotic strategies as another study assessing the antibiotic strategies using a similar study design is underway [13].

### Analysis

The feasibility outcomes of the vanguard study were summarized by treatment groups as proportions. All patients undergoing cardiac surgery during the time of randomization to one of the study arms were included (intention-to-treat, ITT). The adherence to the antibiotic regimen was assessed by reviewing health records in a minimum of randomly selected 5% of eligible patients.

Baseline data are reported by treatment group using count and percentage for categorical data and means and standard deviations for continuous data. Hierarchical modelling (generalized logistic mixed model) was used for the clinical outcomes in the intention-to-treat high-risk patient population with diabetes mellitus or obesity BMI > 30 kg/m^2^. Comparisons between the groups were reported as odds ratio with 95% confidence interval. Due to only two included sites in the vanguard study, the models included centers as random effects to account for the correlation within a center (ICC) and adjusted for the factorial allocation as a fixed effect. As a sensitivity analysis, we assessed the heterogeneity of the treatment effect between the two centers by examining the interaction between treatment and centers. In addition, given the lower-than-expected adherence to Prevena, we conducted a post-hoc as-treated analysis for the primary clinical outcome.

The agreement between the outcome adjudication committee and the site diagnosis for deep/organ s-SSI or all s-SSI, respectively, was calculated using Cohens kappa (κ). The agreement within the 3-member adjudication committee was calculated as the proportion of infections with unanimous agreement among adjudicators out of all deep/organ or superficial s-SSI infections, respectively.

## Results

### Patients

**Fig 1:**
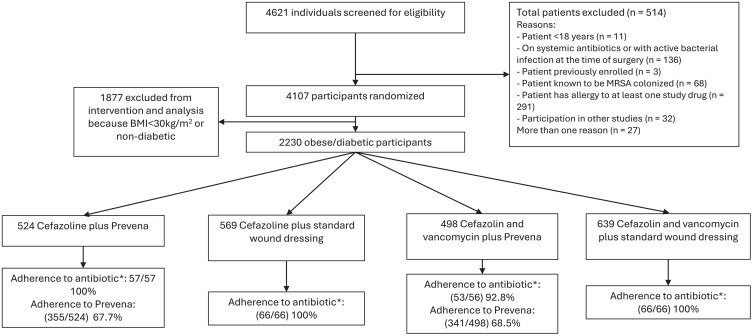
Screening, randomized treatment allocation, and adherence to interventions. Two hospitals were randomly assigned to one of eight potential sequences of the four study arms. 2230 diabetic or obese (BMI > 30 kg/m^2^) underwent cardiac surgery while one of these arms was implemented. *For adherence to antibiotic a total of 245/4107 (6.0%) CRFs were assessed as defined in the statistical analysis plan (Supplementary).

During the study period from April 3, 2018, to December 13, 2022 (04/2018-10/2020 at site 1, and 04/2018-12/2022 at site 2), 4621 patients underwent surgery at the two study centers. A total of 4107/4621 (88.9%) were eligible (Fig 1). Of these, 2230/4107 (54%) were either obese or had diabetes and were eligible for the comparison of the two wound management strategies. A total of 1022/2230 (46%) underwent their cardiac surgery while Prevena was standard of care, and 1208/2230 (54.2%) during a standard wound dressing period (Fig 1).

Of those eligible patients, the overall mean ±standard deviation (SD) age was 67.0 ± 10.7 years and 1025/4107 (25%) were female. Mean ±SD BMI was 29.6 ± 6.1 kg/m^2^. The most common type of surgery was coronary artery bypass grafting (CABG) (n = 2451/4107, 60%) and surgical valve intervention (n = 645/4107, 16%) followed by combined CABG and valve intervention (n = 466/4107, 11%) and other, non-specified interventions (n = 545/4107, 13%). Open vein harvesting on the leg was performed in 1295/4107 (31%) patients. Baseline characteristics are shown in Table 1.

**Table 1:** Demographic and clinical characteristics of the patients at baseline.

	Overall	High Risk (Diabetes or BMI > 30)	Prevena	Standard Care
**Included patients, n**	4107	2230	1022	1208
**Age, mean (SD)**	67.0 (10.7)	66.4 (10.1)	66.3 (9.8)	66.5 (10.4)
**Gender – Female, n (%)**	1025 (25.0)	576 (25.8)	260 (25.4)	316 (26.2)
**BMI (kg/m²), mean (SD)**	29.6 (6.1)	32.8 (6.3)	32.9 (6.0)	32.7 (6.5)
**Comorbidities**				
**Diabetes, n (%)**	1373 (33.4)	1373 (61.6)	632 (61.8)	741 (61.3)
**Renal replacement therapy pre-op, n (%)**	77 (1.9)	56 (2.5)	20 (2.0)	36 (3.0)
**COPD, n (%)**	339 (8.3)	191 (8.6)	86 (8.4)	105 (8.7)
**Peripheral vascular disease, n (%)**	187 (4.6)	120 (5.4)	57 (5.6)	63 (5.2)
**Surgical Procedure**				
**Type of surgery**				
**CABG only, n (%)**	2451 (59.7)	1402 (62.9)	654 (64.0)	748 (61.9)
**Valve only, n (%)**	645 (15.7)	338 (15.2)	146 (14.3)	192 (15.9)
**CABG and Valve, n (%)**	466 (11.3)	247 (11.1)	106 (10.4)	141 (11.7)
**Other, n (%)**	545 (13.3)	243 (10.9)	116 (11.4)	127 (10.5)
**Minimally invasive surgery, n (%)**	43 (1.0)	19 (0.9)	5 (0.5)	14 (1.2)
**Use of bilateral mammary artery, n (%)**	201 (4.9)	101 (4.5)	45 (4.4)	56 (4.6)
**Vein Harvesting, n (%)**	2764 (67.3)	1555 (69.7)	704 (68.9)	851 (70.4)
**Vein Harvesting – open, n (%)**	1295 (31.5)	694 (31.1)	335 (32.8)	359 (29.7)

BMI= Body Mass Index, COPD= Chronic obstructive pulmonary disease, CABG = Coronary artery bypass graft surgery, SD = standard deviation.

### Feasibility outcome

The overall loss to follow-up rate at 90 days was 3.7% (153/4107; 95%CI 3.2–4.3%) and 3.6% (81/2230; 95%CI 2.9–4.4%) for the diabetic or obese population. The adherence to the use of Prevena in eligible patients during study periods when Prevena was considered standard of care was 68.1% (696/1022; 95%CI: 65.2–71.0%) overall, 42.8% (217/507; 95%CI: 38.5–47.1%) for Site A, and 93.0% (479/515; 95%CI: 90.8–95.2%) for site B. Baseline characteristics of non-adherent patients are shown in S1 Table. For compliance with the antibiotic regimen, 245 records of all 4107 (6.0%) randomized patients were evaluated. Documented non-adherence to preoperative, intraoperative, and postoperative antibiotic prophylaxis was rare with 1.2% (95%CI 0.00–2.6%), 0.4% (95%CI 0.00–1.2%), and 0%, respectively.

### Clinical outcomes

The primary clinical outcome, the composite of deep or organ-space s-SSI, occurred in 33/2107 (1.5%) diabetic or obese patients, whereby 16/1022 infections (1.6%) were recorded in the Prevena and 17/1208 (1.4%) in the standard wound dressing group (OR: 1.11; 95% CI: 0.56–2.20) (Table 2). When including superficial infections, a total of 80/2107 (3.6%) s-SSIs were documented, 39/1022 (3.8%) in the Prevena and 41/1208 (3.4%) in the standard wound dressing group (OR:1.12; 95% CI: 0.71–1.75). Among patients who underwent an open venous saphenous harvest during their cardiac surgery, 5/1022 (1.5%) in the Prevena group and 4/1208 (1.1%) in the standard wound dressing period developed an SSI in their leg. The incidence of sternal wound dehiscence was 22/1022 (2.2%) in the Prevena group and 24/1208 (2.0%) in the standard dressing group (OR:1.09; 95% CI: 0.60–1.95). Pain on day 5, length of ICU and hospital stay, acute kidney injury and all-cause mortality in patients with active infection within 90 days were also similar in both groups.

**Table 2:** Outcomes.

	High Risk (Diabetes or BMI > 30)	Prevena	Standard Care	OR (95%CI)/ Mean Diff(95%CI)
**Included patients, n**	2230	1022	1208	
**Primary Clinical Outcome**				
**Deep and/or organ-space s-SSI, n (%)**	33 (1.5)	16 (1.6)	17 (1.4)	1.11 (0.56- 2.20)
**Secondary Clinical Outcomes**				
**All s-SSI including superficial incisional infections, n (%)**	80 (3.6)	39 (3.8)	41 (3.4)	1.12 (0.71- 1.75)
SSIs on the leg*, n (%)	9 (1.3)	5 (1.5)	4 (1.1)	1.34 (0.36- 5.05)
**Wound dehiscence (sternal), n (%)**	46 (2.1)	22 (2.2)	24 (2.0)	1.09 (0.60- 1.95)
**Lab. confirmed *C. difficile* infection, n (%)**	10 (0.4)	6 (0.6)	4 (0.3)	1.78 (0.50- 6.32)
**Mortality in patients with an active infection, n (%)**	2 (0.1)	0 (0.0)	2 (0.2)	
**Length of ICU, mean (SD) [days]**	2.4 (4.4)	2.4 (3.7)	2.5 (5.0)	−0.13 (−0.50- 0.24)
**Length of hospital stay, mean (SD) [days]**	8.3 (10.5)	8.5 (12.2)	8.2 (8.7)	0.35 (−0.52- 1.22)
Acute kidney injury (AKI) within 7 days of surgery, n (%)**	308 (13.8)	139 (13.6)	169 (14.0)	0.94 (0.73- 1.20)
**Stage 1**	207 (9.3)	88 (8.6)	119 (9.9)	
**Stage 2**	72 (3.2)	39 (3.8)	33 (2.7)	
**Stage 3**	29 (1.3)	12 (1.2)	17 (1.4)	
**No AKI**	1795 (80.5)	843 (82.5)	952 (78.8)	
**Missing**	127 (5.7)	40 (3.9)	87 (7.2)	
**Mortality, n (%)**	96 (4.3)	39 (3.8)	57 (4.7)	0.79 (0.52- 1.20)
**Loss of follow up**	81 (3.6)	52 (5.1)	29 (2.4)	2.19 (1.38- 3.47)

s-SSI = sternal surgical site infection, ICU = intensive care unit, SD = standard deviation, OR= odds ratio, 95%CI = 95% confidence interval.

* For patients with open venous saphenous harvest.

**AKI is a percentage increase in serum creatinine of more than or equal to 50%. Stage 1: increase in serum creatinine to more than 150% to 200%. Stage 2: increase in serum creatinine to more than 200% to 300%. Stage 3: increase in serum creatinine to more than 300%.

There was no evidence of interaction between treatment effect and centers (p = 0.905) in the sensitivity analysis. Similar odds ratios for the primary clinical outcome were obtained when excluding patients who did not receive Prevena due to protocol deviations (i.e., post-hoc per-protocol analysis) (deep/organ-space SSI: Prevena n = 11 (1.6%) vs standard dressing n = 17 (1.4%); OR: 1.17; 95% CI 0.54–2.53). A total of ten serious adverse device effects were reported, none of which was classified as device related (S2 Table).

### Agreement of diagnosis

The agreement between the blinded outcome adjudication committee and the site was substantial for deep/organ s-SSI (κ: 0.7, 95% CI: 0.56–0.83) and all s-SSI (κ: 0.63, 95% CI: 0.55–0.71). Agreement for deep/organ s-SSI within the adjudication committee was 100% (33/33) and 47% (22/47) for superficial infections, whereby in 88% (22/25) of the disagreements one reviewer voted for no infection and in the remaining three cases more information was requested.

## Discussion

The *Prevention of Infections in Cardiac Surgery -PREVENA Vanguard study* demonstrates the challenges of a 2x2 factorial crossover design investigating the implementation of new infection prevention interventions as standard of care for s-SSI prevention. Despite meeting the targeted feasibility thresholds for loss to follow up (<10%) and adherence to antimicrobial prophylaxis as per protocol (>90%), the compliance to use the negative pressure wound therapy system Prevena was 68.1%, only, and failed to reach our 90% target.

We observed two contrasting patterns in adherence to the two interventions. Documented non-adherence to antibiotic intervention within the trial was low for each of the time periods and at both study sites. However, the adherence to initiate Prevena in 68.1% did not achieve our predefined target of 90%, highlighting the difficulty in introducing a novel intervention to existing, highly regimented workflows, as present in cardiac surgery. Non-adherence to Prevena was mainly driven by missing application of the wound dressing and not early discontinuation. The fact that one of the two sites showed a much better adherence (93.0% vs 42.8%), may be explained by the fact that this site had experience with using negative pressure wound dressing systems before the launch of the trial, whereas this was a new intervention at the other site. Another major complicating factor was that in contrast to the antibiotic regimen, only the subgroup of patients who were diabetic or obese qualified for the application of Prevena during the respective study period, which required a reliable process to identify those who qualify for Prevena pre-operatively. Again, one site was more successful in implementing this pre-identification process than the other site. Including a longer run-in period to expose sites prior to the change of study arm, might have led to increased adherence. Available comparisons of our adherence rates to similar trials in the literature are limited to gain further insights and adopt possible solutions. Recently published reviews that investigated negative pressure wound therapy did not report utilization of cluster RCTs [11,17,18]. Only one of the included individual-level RCTs – a pilot trial – provided adherence data, showing that 2/35 patients allocated to negative pressure wound dressing were switched to control due to the size of the incision [19]. The learning from the experience with this cluster cross-over pilot/vanguard was that subgrouping a population as part of the study design cannot be reliability implemented, that the protocol must be simple with the same intervention being applied to all patients undergoing the procedure during a given study period, and that for a complex intervention such as the use of a novel wound dressing technology longer phase-in periods may be required.

Regardless of whether data collection dovetailed on the existing routine surveillance conducted by infection prevention and control professionals at one site or a research team at the other site, the trial demonstrated high follow up rates of >95% at the three-month mark.

We used this study design to maximise generalizability in an area of clinical equipoise, to maintain feasibility despite assessing two interventions at the same time, and to reduce the costs of running a large trial with a small event rate. While following the standard of a cluster randomized trial [20], the research questions and study design meet all conditions proposed by the Council of Medical Sciences to obtain a waiver of informed consent [21], and therefore, the study was approved by the Provincial Ethics Board without the requirement for individual patient consent (Clinical Trials Ontario).

This vanguard study was not powered to draw conclusions on clinical outcomes, and therefore, no statistically significant differences in clinical outcomes between the two wound dressing strategies were expected. Sensitivity analysess investigating heterogeneity of the treatment effect by site or only including on-treatment patients to account for the low rate of adherence to Prevena, did not show a statistically significant difference either.

Generally, the assessment of SSI can be challenging, and correct interpretation of case vignettes varies [22,23]. Agreement among blinded adjudicators for deep and organ-space s-SSIs was previously shown to be high [24], and was successfully demonstrated in our vanguard study. Blinded adjudication may be important to overcome the risk of bias from subjective outcomes in unblinded trials [25]. In this pilot/vanguard, there was unanimous agreement on the primary outcome among the outcome adjudication committee members, and the agreement between sites and the adjudication committee was substantial and similar to previous reports for deep infection [24]. Disagreement within the adjudication committee was noticed for superficial infections, which supports the choice of deep/organ-space infections as a primary outcome given the higher level of subjectivity for superficial incisional infections.

This vanguard trial has several limitations. First, the trial was stopped after the vanguard phase due to lack of additional funding, hence, it was not powered to draw conclusions on treatment effects. Second, even if most probable of negligible relevance, the trial was repeatedly paused during the COVID-19 pandemic at one site, prolonging the recruitment phase. Reasons for the interruption were lack of study staff or outstanding Prevena resupply during the pandemic and awaiting a protocol amendment to increase recruitment number at the site.

In conclusion, conducting the study as planned with a 2x2 factorial cross-over design was feasible. However, adherence to one of the interventions, the Prevena negative wound management system, was lower than expected and suggested that an eventual full trial needs to be simplified with all patients regardless of their individual risk factors to be managed the same way in a given study period.

## Supporting information

S1 Fig Study design. (PDF)

S1 Table Baseline characteristics of patients adherent to Prevena vs. non-adherent. (PDF)

S2 Table Serious Adverse Device Effect (SADE). (PDF)

S1 Appendix Protocol. (PDF)

S2 Appendix Consort checklist. (PDF)

S3 Appendix Statistical analysis plan. (PDF)

S4 Appendix Study team. (PDF)

S5 Appendix Dataset. (CSV)

S6 Appendix Codebook. (TXT)

S1 File Study protocol. (PDF)
