# Supplementary figures and images for: Prevention of Infections in Cardiac Surgery (PICS)-Prevena Study – A pilot/vanguard factorial cluster cross-over RCT
Source: PLoS One. 2025 Dec 15;20(12):e0338300. doi: 10.1371/journal.pone.0338300 (PMC12704892; doi:10.1371/journal.pone.0338300)

Supplementary Figure 1: Study design

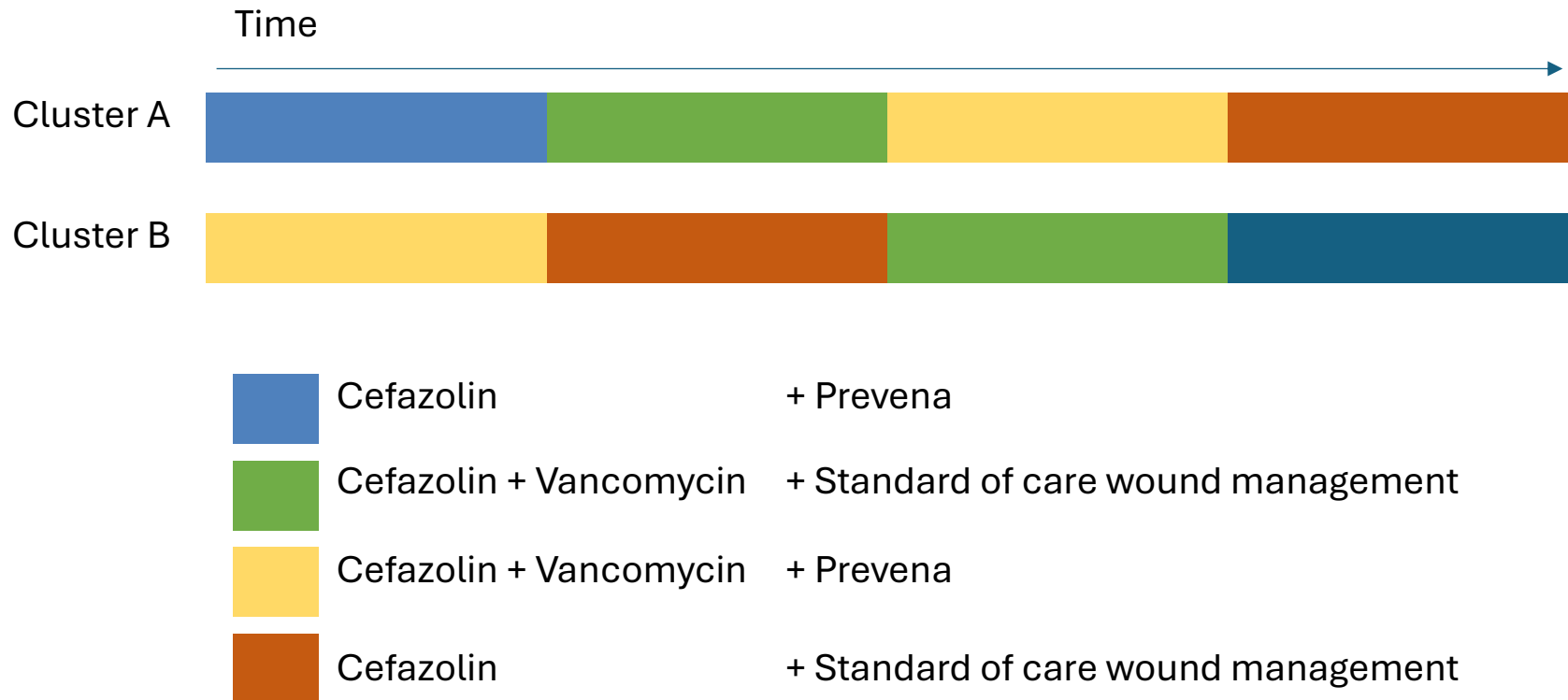

Supplement: S1 Fig — (PDF) [file pone.0338300.s001.pdf]
