# Supplementary material for: Prevention of Infections in Cardiac Surgery (PICS)-Prevena Study – A pilot/vanguard factorial cluster cross-over RCT
Source: PLoS One. 2025 Dec 15;20(12):e0338300. doi: 10.1371/journal.pone.0338300 (PMC12704892; doi:10.1371/journal.pone.0338300)
Supplement: S1 Table — (PDF) [file pone.0338300.s002.pdf]

Supplementary Table 1: Baseline characteristics of patients adherent to Prevena vs. non-adherent

	High Risk (Diabetes and/or BMI>30)	Randomized to Prevena	Received Prevena	Not Received Prevena
**N Randomized and eligible**	2230	1022	696	326
**Age, mean (SD)**	66.4 (10.1)	66.3 (9.8)	66.7 (9.6)	65.5 (10.0)
**Gender - Female, n (%)**	576 (25.8)	260 (25.4)	179 (25.7)	81 (24.8)
**BMI, mean (SD)**	32.8 (6.3)	32.9 (6.0)	33.0 (6.0)	32.6 (5.9)
**Comorbidities**				
**Diabetes, n (%)**	1373 (61.6)	632 (61.8)	437 (62.8)	195 (59.8)
**Renal replacement therapy pre-op, n (%)**	56 (2.5)	20 (2.0)	14 (2.0)	6 (1.8)
**COPD, n (%)**	191 (8.6)	86 (8.4)	50 (7.2)	36 (11.0)
**Peripheral vascular disease, n (%)**	120 (5.4)	57 (5.6)	31 (4.5)	26 (8.0)
**Surgical Procedure**				
**Type of surgery**				
**CABG only, n (%)**	1402 (62.9)	654 (64.0)	470 (67.5)	184 (56.4)
**Valve only, n (%)**	338 (15.2)	146 (14.3)	84 (12.1)	62 (19.0)
**CABG and Valve, n (%)**	247 (11.1)	106 (10.4)	62 (8.9)	44 (13.5)
**Other, n (%)**	243 (10.9)	116 (11.4)	80 (11.5)	36 (11.0)
**Minimally invasive surgery, n (%)**	19 (0.9)	5 (0.5)	4 (0.6)	1 (0.3)
**Use of bilateral mammary artery, n (%)**	101 (4.5)	45 (4.4)	40 (5.7)	5 (1.5)
**Vein Harvesting, n (%)**	1555 (69.7)	704 (68.9)	477 (68.5)	227 (69.6)
**Vein Harvesting - open, n (%)**	694 (31.1)	335 (32.8)	139 (20.0)	196 (60.1)
