# Supplementary material for: Prevention of Infections in Cardiac Surgery (PICS)-Prevena Study – A pilot/vanguard factorial cluster cross-over RCT
Source: PLoS One. 2025 Dec 15;20(12):e0338300. doi: 10.1371/journal.pone.0338300 (PMC12704892; doi:10.1371/journal.pone.0338300)
Supplement: S2 Table — (PDF) [file pone.0338300.s003.pdf]

Supplementary Table 2: Serious Adverse Device Effect (SADE)

	High Risk (Diabetes or BMI>30)	Prevena	Standard Care
**Randomized and eligible, N**	2230	1022	1208
**Patients with SADE, N (%)**	10 (0.4)	10 (1.0)	0 (0.0)
**Device related, N**	0	0	0 (0.0)
**Outcome**			
- **Recovered with sequelae, N (%)**	1 (0.0)	1 (0.1)	0 (0.0)
- **Fatal, N(%)**	9 (0.4)	9 (0.9)	0 (0.0)
**Cardiac arrest, N (%)**	2 (0.1)	2 (0.2)	0 (0.0)
**Cardiogenic shock, N (%)**	2 (0.1)	2 (0.2)	0 (0.0)
**Multiple organ dysfunction syndrome, N (%)**	1 (0.0)	1 (0.1)	0 (0.0)
**Post procedural haemorrhage, N (%)**	0 (0.0)	0 (0.0)	0 (0.0)
**Neck injury, N (%)**	1 (0.0)	1 (0.1)	0 (0.0)
**Vasoplegia syndrome, N (%)**	1 (0.0)	1 (0.1)	0 (0.0)
**Acute respiratory failure, N (%)**	1 (0.0)	1 (0.1)	0 (0.0)
**Aortic dissection, N (%)**	1 (0.0)	1 (0.1)	0 (0.0)
**Missing, N (%)**	1 (0.0)	1 (0.1)	0 (0.0)
