# Supplementary material for: Prevention of Infections in Cardiac Surgery (PICS)-Prevena Study – A pilot/vanguard factorial cluster cross-over RCT
Source: PLoS One. 2025 Dec 15;20(12):e0338300. doi: 10.1371/journal.pone.0338300 (PMC12704892; doi:10.1371/journal.pone.0338300)
Supplement: S1 Appendix — (PDF) [file pone.0338300.s004.pdf]

DocuSign Envelope ID: 2F66E700-BE3D-413C-BA9A-47B956666541

PREVENTION OF INFECTIONS IN CARDIAC SURGERY (PICS) PREVENA VANGUARD STUDY:

A CLUSTER-RANDOMIZED FACTORIAL CROSSOVER TRIAL, COMPARING ANTIBIOTIC MONO-PROPHYLAXIS WITH CEFAZOLIN VS. DUAL-PROPHYLAXIS WITH CEFAZOLIN PLUS VANCOMYCIN AND CONVENTIONAL WOUND DRESSING VS. PREVENA NEGATIVE-PRESSURE WOUND MANAGEMENT

Protocol Version 4.0, 2022-08-08

Principal Investigator:
Dr. D. Mertz, MD, MSc
Juravinski Hospital and Cancer Center
711 Concession Street, Section M, Level 1, Room 3
Hamilton, ON, Canada, L8V 1C3

Co-Investigators:

Dr. P.J Devereaux, MD, PhD, FRCPC<br>Population Health Research Institute<br>Hamilton General Hospital, DBCVSRI<br>237 Barton Street East<br>Hamilton, Ontario, Canada L8L 2X2	Dr. Stuart Connolly, MD, FRCPC<br>Population Health Research Institute<br>Hamilton General Hospital, DBCVSRI<br>237 Barton Street East<br>Hamilton, Ontario, Canada L8L 2X2
Dr. Andre Lamy, M.Sc. MD, FRCS(C)<br>Population Health Research Institute<br>Hamilton General Hospital, DBCVSRI<br>237 Barton Street East<br>Hamilton, Ontario, Canada L8L 2X2	Dr. Mark Loeb, BSc, MD, MSc, FRCPC<br>McMaster University<br>1200 Main Street West<br>Hamilton, Ontario, Canada L8N 3Z5
Dr. Michael McGillion, RN, PhD<br>Population Health Research Institute<br>Hamilton General Hospital, DBCVSRI<br>237 Barton Street East<br>Hamilton, Ontario, Canada L8L 2X2	Dr. Michael Stacey, MBBS DS W. Aust. FRACS<br>Hamilton General Hospital – 6 North<br>237 Barton Street East<br>Hamilton, Ontario, Canada L8L 2X2

Steering Committee Chair:
Dr. R. Whitlock, MD, PhD, FRCSC
Population Health Research Institute, DBCVSRI
237 Barton St. E., Room 1C1-5B
Hamilton, ON, Canada, L8L 2X2

DocuSign Envelope ID: 2F66E700-BE3D-413C-BA9A-47B956666541

Sponsor and Study Coordinating Group:
Population Health Research Institute
Hamilton General Hospital, DBCVSRI
237 Barton St. E., Room C1-237
Hamilton, ON, Canada, L8L 2X2

Funding:
Acelity

DocuSign Envelope ID: 2F66E700-BE3D-413C-BA9A-47B956666541

This protocol (Version 4.0 2022-08-08) has been developed by the Principal Investigator, Co-Investigators, and Steering Committee members and its contents are the intellectual property of this group. It is an offence to reproduce or use the information and data in this protocol for any purposes other than the PICS Prevena Study without prior approval from the Steering Committee.

SIGNATURES

Dr. Dominik Mertz<br>Principal Investigator<br>Population Health Research Institute	DocuSigned by:<br>Dr. Dominik Mertz<br>0EFAE0B7616444E...<br>Signature	2022-Aug-09 | 08:04 EDT<br>Date (YYYY-MM-DD)
Dr. Richard Whitlock<br>Steering Committee Chair<br>Population Health Research Institute	DocuSigned by:<br>Dr Richard Whitlock<br>933778A89E0347E...<br>Signature	2022-Aug-08 | 12:45 EDT<br>Date (YYYY-MM-DD)

STUDY CONTACTS

**Investigator**	Name:	Dr. Dominik Mertz
	Address:	Juravinski Hospital and Cancer Center<br>711 Concession Street, Section M, Level 1, Room 3<br>Hamilton, ON, Canada, L8V 1C3
	Tel:	(905) 527 4322 ext. 43952
	Email:	mertzd@mcmaster.ca

**Project Officer**	Name:	Dr. Sarah Khan
	Address:	Division of Infectious Diseases, Department of Pediatrics, McMaster University, 1280 Main Street West, HSC 3A, Hamilton, ON, L8S 4K1
	Email:	Khan259@mcmaster.ca

**Project Manager**	Name:	Ms. Jessica Vincent
	Address:	Population Health Research Institute<br>237 Barton Street East, Hamilton, ON, L8L 2X2
	Tel:	(905) 527 4322 ext. 40635
	Email:	jessica.vincent@phri.ca

**Study Coordinator**	Name:	Ms. Ingrid Copland
	Address:	Population Health Research Institute<br>237 Barton Street East, Hamilton, ON, L8L 2X2
	Tel:	(905) 527 4322 ext. 40368
	Email:	ingrid.copland@phri.ca

**Project Office Email**	prevena@phri.ca

TABLE OF CONTENTS

1.0 PROTOCOL SYNOPSIS 8
2.0 INTRODUCTION 11
3.0 BACKGROUND AND RATIONALE 12
4.0 STUDY GOALS / OBJECTIVES 13
5.0 STUDY DESIGN AND METHODOLOGY 13
5.1 STUDY DESIGN 13
5.2 INCLUSION AND EXCLUSION CRITERIA 14
5.3 INTERVENTIONS 15
5.3.1 ANTIBIOTIC PROPHYLAXIS 15
5.3.2 WOUND MANAGEMENT 15
5.4 STUDY PROCEDURES 16
5.4.1 RANDOMIZATION 16
5.4.2 BLINDING AND TREATMENT SUPPLY 16
5.4.3 DATA COLLECTION AND REPORTING 17
5.5 STUDY OUTCOME EVENTS 17
5.5.1 PROPOSED VANGUARD STUDY 17
5.5.2 EVENTUAL FULL TRIAL 18
5.5.2.1 PRIMARY OUTCOME 18
5.5.2.2 SECONDARY OUTCOMES 18
5.5.3 OUTCOME ADJUDICATION IN THE EVENTUAL FULL TRIAL 18
5.6 SAMPLE SIZE AND STATISTICAL METHODS 20
5.6.1 SAMPLE SIZE VANGUARD STUDY 20
5.6.2 SAMPLE SIZE CALCULATION EVENTUAL FULL TRIAL 20
5.6.3 ANALYSIS PLAN 21
6.0 ADMINISTRATIVE AND REGULATORY 22
6.1 ETHICS REVIEW 22
6.3 TIMELINES AND MILESTONES 22
6.4 DATA SAFETY AND MONITORING BOARD 23

DocuSign Envelope ID: 2F66E700-BE3D-413C-BA9A-47B956666541

6.5 STATEMENT OF CONFIDENTIALITY 23
6.6 AUDIT AND INSPECTION 24
6.7 ROLE OF THE INDUSTRY SPONSOR 24
7.0 SCIENTIFIC REPORTING AND PUBLICATION 24
8.0 REFERENCES 25
APPENDIX A 31

DocuSign Envelope ID: 2F66E700-BE3D-413C-BA9A-47B956666541

List of Abbreviations

BMI: Body Mass Index
CANNeCTIN: Canadian Network and Centre for Trials Internationally
DSMB: Data Safety Monitoring Board
FDA: Food and Drug Administration
ICC: Intra-class Correlation
IPC: Inter-period Correlation
ICU: Intensive Care Unit
IPAC: Infection Prevention and Control
MRSA: Methicillin-resistant S. aureus
NHSN/CDC: National Healthcare Safety Network/Centres for Disease Control
OR: Operating Room
PADIT: Prevention of Arrhythmia Device Infection Trial
PHRI: Population Health Research Institute
PIMS: Prevena Incision Management System
RCT: Randomized Controlled Trials
s-SSI: Sternal Surgical Site Infection
VAS: Visual Analog Scale
WHO: World Health Organization

DocuSign Envelope ID: 2F66E700-BE3D-413C-BA9A-47B956666541

1.0 PROTOCOL SYNOPSIS

TITLE OF STUDY	Prevention of Infections in Cardiac Surgery (PICS) Prevena Study: A Cluster-Randomized Factorial Crossover trial
FUNDING	Acelity
PRINCIPAL INVESTIGATOR	Dominik Mertz, MD, MSc
COORDINATING CENTER	Population Health Research Institute (PHRI) of Hamilton Health Sciences and McMaster University
CLINICAL/REGULATORY PHASE	Phase IV
PARTICIPATING CENTRES	The vanguard study will be conducted at two study sites in Canada. The eventual full trial will be conducted at 19 clinical centers in Canada and other countries.
STUDY OBJECTIVE	The main objective of this vanguard study is to test the feasibility of the study design.<br>The primary goal of the eventual full trial is to determine the effects of dual antibiotic prophylaxis and of a negative-pressure wound management system (Prevena) on sternal surgical site infections in patients undergoing cardiac surgery.
STUDY DESIGN	Multi-center, factorial, cluster crossover study. Centers will be randomized to one of eight orders of the four study arms: 1) cefazolin prophylaxis plus Prevena*(*diabetic and/or obese patients (BMI >30kg/m^2)) 2) cefazolin and vancomycin prophylaxis plus Prevena*(*diabetic and/or obese patients (BMI >30kg/m^2)), 3) cefazolin prophylaxis plus standard wound dressing, 4) cefazolin and vancomycin prophylaxis plus standard wound dressing. Each study arm will be implemented for 4-8 months (aiming for roughly 500 patients per arm and site), and the strategy will become standard of care for all patients undergoing surgery during that time period. A wash-in period of one month prior to each arm will allow for the transition in management strategies.<br>*Note: If due to an unforeseen circumstance (e.g. COVID-19) where surgeries may stop or be reduced, the study arm may be extended. The new timeline for the study arm will need approval by the study Principal Investigator.*
NUMBER OF PATIENTS	Two study centers and a total of roughly 4,000 patients for the vanguard study, i.e. 500 patients per arm at each center. For the eventual full trial, an additional 17 study centers and 32,334 patients will be needed

DocuSign Envelope ID: 2F66E700-BE3D-413C-BA9A-47B956666541

STUDY DURATION	Study duration at each site depends on the volume of surgeries. For an average sized site it is 31 months: 4x6 months for the four study arms, 4x1 month for the phase-in, and 3 months follow-up after completion of study enrolment.
INCLUSION CRITERIA	≥18 years of age undergoing open-heart surgery (sternotomy, including minimally-invasive sternotomies)
EXCLUSION CRITERIA	1. On systemic antibiotics or with an active bacterial infection at the time of surgery<br>2. Patients previously enrolled in this trial<br>3. Patients known to be colonized with MRSA (unethical not to administer glycopeptides), beta-lactam or vancomycin allergy precluding the use of cefazolin or vancomycin, respectively, or to silver precluding the use of Prevena<br>4. Participation in other studies that may interfere with this trial
PATIENTS INCLUDED IN ANALYSIS	All eligible patients during the study period. Only obese and/or diabetic patients will be analysed for the Prevena versus standard wound dressing comparison.
PRIMARY OUTCOME VANGUARD STUDY	1. Adherence to the wound management system as per protocol<br>2. Adherence to the antibiotic regimen as per protocol<br>3. Loss of follow-up
PRIMARY OUTCOME EVENTUAL FULL TRIAL	Deep and/or organ-space sternal surgical site infection (s-SSI) using NHSN/CDC definitions
SECONDARY OUTCOMES EVENTUAL FULL TRIAL	1. All s-SSI including superficial incisional infections<br>2. SSIs on the leg (in patients with open venous saphenous harvest)<br>3. Wound dehiscence<br>4. Laboratory confirmed *C. difficile* infection<br>5. Mortality in patients with an active infection.<br>6. Length of ICU- and hospital stay<br>7. Pain on day 5 +/- 1 day (visual analog scale, VAS)<br>8. Acute kidney injury within 7 days of surgery
INTERVENTIONS	**Antibiotic mono-prophylaxis:**<br>Cefazolin 2g (or 3g if greater than 120kg body weight) will be given within an hour of surgery, followed by one intra-operative dose of cefazolin at 4 hours after the first dose or upon wound closure (whatever comes first), and finally two post-operative doses q8h.<br>**Antibiotic dual prophylaxis:** As above plus vancomycin at roughly 15mg/kg body weight intravenously, i.e. 1g, or 1.5g

DocuSign Envelope ID: 2F66E700-BE3D-413C-BA9A-47B956666541

	if greater than 85kg body weight. No intra-operative dose of vancomycin will be given, and a single second dose will be given 12 hours after the first dose.<br>**Standard wound dressing:** non-negative wound dressing as standard of care at the study site.<br>**Prevena Negative-Pressure Wound Management System:** Prevena will be applied to all diabetic and/or obese patients (BMI >30kg/m2) at the end of surgery on the sternal as well as the vein harvest site (if open saphenous vein harvest) in the OR and left in place for 7 days.
RANDOMIZATION	Centers will be randomized to one of eight possible orders of the four study arm. Centers will get informed about the next study arm 4 weeks prior to the implementation of the following arm.
EVALUATION PERIOD	All outcomes will be evaluated 90 days after surgery as per the NHSN/CDC definition for s-SSIs.
ASSESSMENT OF EVENTS	The study sites will prepare case reports of all cases meeting criteria of a potential SSIs for the outcome adjudication committee. The reports will not include any information (e.g. dates) that would allow the blinded committee to guess the study arm of a particular patients.
STATISTICAL ANALYSIS	The primary (feasibility) outcomes of the vanguard study will be analysed as proportions with 95% confidence intervals.<br>For the eventual full trial, we will apply hierarchical modelling (generalized linear mixed model) for the primary analysis in order to adjust for cluster effects, stratified according to the factorial allocation in the intention-to-treat patient population meeting inclusion criteria.
DATA SAFETY AND MONITORING BOARD	An independent data safety and monitoring board will evaluate safety.
ADJUDICATION COMMITTEE	Blinded adjudication of the s-SSIs will be performed by a committee consisting of three members.

DocuSign Envelope ID: 2F66E700-BE3D-413C-BA9A-47B956666541

2.0 INTRODUCTION

Despite the routine use of antibiotics before and after cardiac surgery, infections of the chest wound (sternal surgical site infection, s-SSI) remain a common life-threatening complication of heart surgery. Antibiotic prophylaxis is well accepted as the cornerstone of prevention for these infections; the best choice of antibiotic prophylaxis in patients undergoing open heart surgery is, however, unclear. Roughly 40% of pathogens identified in chest wound infections, such as coagulase-negative staphylococci and methicillin-resistant *S. aureus* (MRSA), are not covered by the recommended cefazolin prophylaxis, hence, there is large variability in practice. On the other hand, the risks associated with unnecessary use of antibiotics are well documented and include the emergence of "superbugs" and *Clostridium difficile* infections. Furthermore, negative pressure wound management systems can reduce the risk of SSIs by sealing the surgical site, removing potentially infected exudates, and improving wound healing. This Health Canada approved technology is showing promising results in small and non-randomized studies in cardiac surgery as well as other disciplines, but there is a lack of high-level evidence to support its use.
The here proposed vanguard of the eventual multi-centre 2x2 factorial cluster randomized cross over trial was designed to test whether dual antibiotic prophylaxis with cefazolin plus vancomycin is superior to cefazolin mono-prophylaxis, and whether a negative-pressure wound management system (Prevena) is superior to standard wound dressing. In a first step, a vanguard study will be launched at two sites to establish feasibility of the study design. The proposed design is similar to the recently completed antibiotic prophylaxis to prevent infection in arrhythmia devices (PADIT) study[1]. Furthermore, we are currently conducting a multi-centre vanguard trial (refered to as ‚current vanguard study' below) at three hospital sites (Hamilton Health Sciences, Hamilton, ON; Sherbrook University, Sherbrooke, QC; St. Michael's Hospital, Toronto, ON) using a very similar design and testing two interventions: mono versus dual antibiotic therapy as in our here proposed trial, and antibiotic duration (pre- and intra-operative doses versus continued prophylaxis post-operatively). To date, we have successfully implemented nine different study arms across all sites and enrolled a total of more than 3,000 patients. Based on 1,046 patients in which adherence to the antibiotic protocol was audited, the pre-operative dose was not given according to the protocol in 3.1% of cases, the intra-operative dose in 2.7%, and the post-operative antibiotics in 2.2% of cases. The combined 7.4% of deaths and/or loss of follow-up for other reasons confirm the figures we have used in our sample size calculation. To date, our *a priori* defined feasibility criteria of the current vanguard study are met. The outcome adjudication process has already been successfully piloted in a pilot study[2].

DocuSign Envelope ID: 2F66E700-BE3D-413C-BA9A-47B956666541

3.0 BACKGROUND AND RATIONALE

Deep s-SSIs are a leading cause of morbidity and mortality. In-hospital mortality in cardiac surgery patients is ~3.6%, and this risk quadruples in patients with s-SSI and healthcare costs are estimated to be $40,000 per s-SSI.[3-8]

Context for choice of antimicrobials

Antibiotic prophylaxis is considered the cornerstone of prevention for s-SSIs, and yet, there is a major gap in knowledge regarding what the best antimicrobial regimen is. Largely due to the lack of evidence around choice and duration of antibiotics[9,10], adherence to guidelines in terms of antibiotic prophylaxis is poor.[11-13]

Current guidelines consider cephalosporins as the first line for prophylaxis in cardiac surgery.[9,14,15] These recommendations are based on observational studies and small randomized-controlled trials (RCTs) that were not sufficiently powered to detect important differences in patient outcomes.[9] Despite these guidelines, up to one third of U.S cardiac surgery centres report using vancomycin routinely, either in combination or alone.[11,12,16] The rationale is that many common pathogens of s-SSIs, coagulase-negative Staphylococci, MRSA and enterococci, are resistant to the recommended antibiotic, cefazolin (a cephalosporin).[15,17-19] Glycopeptides (e.g. vancomycin) with activity against these pathogens may reduce the incidence of s-SSI[16,20,21] without significant harm when used for a short period.[12,22] However, glycopeptides have a number of disadvantages, such as adverse reactions, resistance, cost, may be less efficacious for cefazolin susceptible pathogens, and the potential of acute kidney injury.[23-25] Therefore, rigorous RCT data is needed to move the field forward.[10,26] Given the potential advantage of cephalosporins, such as being well tolerated and highly active against susceptible pathogens, but also the increasing incidence of s-SSIs caused by cephalosporin resistant gram-positive pathogens, there is an important need to generate high quality evidence testing the combination of a cephalosporin and a glycopeptide.[26]

Context for choice of wound management

A complementary approach to the prevention of s-SSI is the optimal management of the surgical wound. Accumulation of bacteria in the surgical site as well as bacteria gaining access to the deeper layers in the postoperative phase may be responsible for the development of SSIs. This may be prevented by the use of negative-pressure management systems that a) seal the surgical site, b) remove potentially infected exudates from the surgical site, and c) improve wound healing by increased blood flow, edema reduction, and mechanical stretching of cells leading to cell growth and expansion.[27,28]

Small and non-randomized studies have shown a benefit of negative-pressure wound management products in cardiac surgery[29] and in other surgical disciplines[30-32] as recently summarized in a systematic review[33], but large, rigorous RCTs are missing in the field. Nevertheless, the recently published World Health Organization (WHO) guideline on prevention of surgical site infections[34] is recommending their use. The WHO guideline panel conducted a systematic review and identified 19 publications with 20 studies (6 RCTs and 14 observational studies) on the use of negative pressure wound therapy. They

DocuSign Envelope ID: 2F66E700-BE3D-413C-BA9A-47B956666541

found a reduction in the odds for SSIs of 0.56 (95% CI 0.32-0.96) in RCTs and 0.30 (95% CI 0.22-0.42) in observational studies. In particular for cardiac surgery the evidence was clearly in favor of using this approach (OR 0.29, 95% CI 0.12-0.69). However, the guideline states that this is a conditional recommendation based on low quality evidence, and as such, large, rigorous and convincing trials are needed.[34]

Rational for the study design

A cluster design is the most appropriate for highly standardized operational procedures as we propose.[1] This design aligns research with clinical care allowing enrolment of a representative patient population in a real-world setting[35] for a comparative effectiveness study while minimizing cross-contamination.[1] The cross over between the two strategies will minimize bias from changes in practice or alterations in the distribution of bacterial pathogens, and also has the advantage of having each centre serve as its own control reducing potential bias from imbalances between sites.[1]

In summary, there is a lack of high quality evidence on how best to prevent s-SSIs in cardiac surgery patients. This has led to variability in practise and non-adherence to guidelines. The proposed study will test whether dual antibiotic prophylaxis is superior to the currently recommended single-agent prophylaxis with cefazolin, and shed light on whether or not modern negative-pressure wound management technology lower the risk of s-SSIs. As such, we anticipate that the eventual full trial will be a landmark trial influencing future clinical practice guidelines and clinical practise in Canada and elsewhere.

4.0 Study Goals / Objectives

The primary goal of the proposed vanguard study is to test the feasibility of the study design (i.e. protocol adherence, loss of follow-up, see below), in particular of the large scale use of Prevena given that the antibiotic arms have already been successfully pilot tested in the current vanguard study.

The primary goal of the eventual full trial is to determine the effects of dual antibiotic prophylaxis and of the Prevena negative-pressure wound management system on s-SSIs in patients undergoing cardiac surgery.

5.0 Study Design and Methodology

5.1 Study Design

The proposed vanguard study will be using the same design as the eventual full trial which is a 2x2 factorial crossover cluster RCT comparing mono- versus dual antibiotic prophylaxis and the Prevena negative-pressure wound management system versus standard wound dressing in patients undergoing cardiac surgery with a primary outcome of deep or organ/space s-SSI.

Hospitals will be randomized to one of eight possible orders of four study arms. Such a cluster RCT offers clear advantages over an individual-level RCT including a reduced risk for contamination of the intervention, a feasible recruitment strategy, substantial reduction in cost and resources, an increased spectrum of participant characteristics (enhancing generalizability), and a simplified consent process. During each trial period, the particular prophylactic regimen and choice of wound management will become the standard for the centre for all patients undergoing cardiac surgery.
The antibiotic regimen as well as the choice of wound management to be used becomes standard of care, however, the surgeons/physicians ordering the antibiotics and wound dressing have the final say and can opt out from this recommendation if they wish to do so. Based on the current vanguard study, this happens very rarely (see above), and missing doses that had been ordered but not administered were the main reason for non-adherence. There will be a phase-in of four weeks to allow enough time for full implementation of the next study arm. This is highly relevant to the antibiotic regimen, as peri-operative antibiotics are frequently being ordered by the surgeon when they see their elective patients pre-operatively. Most patients are undergoing surgery within 4 weeks, thus, a phase-in of 4 weeks prevents that the orders for many patients need to be changed on the day of surgery. For the wound management strategy, such a long phase in is not needed, thus, in order to avoid additional costs, Prevena will be used in the last week of the phase-in, only. Our intention is that the proposed vanguard study data be included as part of the full trial data. We fully understand that there is controversy in the field as to whether this is a proper approach;[36] however, if there are no meaningful changes/differences in the protocol between the proposed vanguard study and the full trial, it is considered acceptable to include the vanguard data.[37]

5.2 INCLUSION AND EXCLUSION CRITERIA

Centres from Canada and elsewhere performing >300 cardiac procedures annually and willing to participate will be eligible. At each centre, all patients undergoing cardiac surgery during each study period will receive the study antibiotic and wound management strategy in place (i.e. standard wound dressing or Prevena in diabetic or obese patients), but only patients fulfilling eligibility criteria will be evaluated in this study.
We will include adult patients (≥18 years of age) undergoing open-heart surgery (sternotomy, including minimally-invasive sternotomies) and of these will exclude patients on antibiotics or with an active bacterial infection at the time of surgery, patients previously enrolled in this trial, patients known to be colonized with MRSA (unethical not to administer glycopeptides), beta-lactam or vancomycin allergy precluding the use of cefazolin or vancomycin, respectively, and participation in other studies that may interfere with this trial. Furthermore, patients with sensitivity to silver will receive standard wound dressing in all study arms as Prevena is contraindicated in these patients.

DocuSign Envelope ID: 2F66E700-BE3D-413C-BA9A-47B956666541

5.3 INTERVENTIONS

There will be four study arms: 1) cefazolin prophylaxis plus Prevena 2) cefazolin and vancomycin prophylaxis plus Prevena, 3) cefazolin prophylaxis plus standard wound dressing, 4) cefazolin and vancomycin prophylaxis plus standard wound dressing.

5.3.1 ANTIBIOTIC PROPHYLAXIS

For patients with normal renal function, cefazolin 2g (or 3g if greater than 120kg body weight) will be given within an hour of surgery.[15] In addition, one intra-operative dose of cefazolin at 4 hours after the first dose or upon wound closure (whatever comes first) will be administered. Finally, two post-operative doses q8h will be given. Vancomycin will be dosed at roughly 15mg/kg body weight intravenously, i.e. 1g or 1.5g if greater than 85kg body weight.[15] No intra-operative dose of vancomycin will be given, and a second dose will be given 12 hours after the first dose.
As the data on the optimal duration of antibiotic prophylaxis in cardiac surgery patients is unclear as previously shown by our group as well as by Lador et al.[9,10], we decided to continue antibiotic prophylaxis for up to 24 hours post-operatively. This best reflects current standard of care in Canada based on our own survey: of 11 centers that responded, 2 centers stopped within 18 hours post-operatively, the majority -4 sites- at the 24 hour mark, and 5 continued up to 48 hours post surgery (unpublished data). The 24 hours are also a compromise between the recommendation in published guidelines that vary with recommended durations ranging from one single dose to up to 72 hours after surgery.[6,9,10,15,34,38-41] A third rationale for not using prophylaxis beyond 24 hours is based on evidence that a longer duration may increase the risk for infections by resistant pathogens[42] and may increase the non-SSI infection risk as well as *C. difficile* infection.[12,43]

5.3.2 WOUND MANAGEMENT

We will be using one of the approved and marketed negative-pressure wound management systems, the Prevena Incision Management System (PIMS). This system is being used in many surgical disciplines and has been used in studies in cardiac surgery at the sternal as well as the saphenous vein harvest site in the past.[29,44,45] Prevena will be applied to sternal wounds as well as leg wounds in patients with saphenous vein harvest for coronary artery bypass surgery. Given the costs of such wound management technologies, only the subgroup of patients deemed to be at increased risk for s-SSIs will be managed with this technology, i.e. patients with either diabetes mellitus and/or obesity defined as BMI of 30 or greater. Furthermore, it will be applied to leg incision sites only in patients who underwent an open saphenous vein harvesting procedure, i.e. Prevena will not be used in patients in whom an endoscopic approach was used which had been shown to result in significantly lower infection rates by a factor 4-5 compared to open surgery.[46-48] This is in keeping with the recently published WHO guideline on prevention of surgical site

DocuSign Envelope ID: 2F66E700-BE3D-413C-BA9A-47B956666541

infections where negative pressure wound management systems are recommended for at risk patients.[34] Diabetes mellitus was found to increase the risk for s-SSI in the range of 2.1-4.2 times and obesity by 1.8-6.6 times.[33,49-54]

The device is for single patient use, battery-powered, disposable, and delivers continuous 125 mmHg negative pressure to the closed surgical incision for a 7-day therapy period. It is an easy to use device that also provides audible and visual alerts for low battery, maximum canister volume, and leak conditions. Additional alerts include system error and device life-cycle expiration (8 days). It is contained in a water-resistant housing, which allows the subject to lightly shower with the device. Wound fluids are contained within the 45 mL canister. We will be using the PIMS Peel and Place Dressing which is a multi-layer dressing that is applied to the surgically closed wound, without sizing or cutting. The product is FDA and Health Canada approved for use on surgical sites. It should be used with caution on patients with fragile skin surrounding the incision and in subjects who are on anticoagulants. Instructions for use as well as all user manuals will be provided with the study product.

Any non-negative pressure wound dressing that is routinely used at study sites will be considered standard of care in the control arms.

5.4 STUDY PROCEDURES

5.4.1 RANDOMIZATION

Centres will be allocated to one of eight randomly selected orders with each of the four treatments arms equally represented. Both arms of each factor need to be represented in the first two and second two study arms to allow a cross over from the first to the second set of study arms for both factors (ADBC, ADCB, DABC, DACB, BCAD, BCDA, CBAD, CBDA). We recognise that cluster RCTs over prolonged periods of time are at risk of confounding through changes over time (e.g. changes in the epidemiology or resistance profiles of pathogens). The proposed cross over design will however minimize this risk while not adversely affecting study power. In order to reduce the risk of allocation bias, the local study teams will only be informed about the next arm they are randomized to 4 weeks prior to the switch to the next arm.

We will randomize all sites for the eventual full trial upfront, and then apply the allocation for site 1 and site 2 for the proposed vanguard study.

5.4.2 BLINDING AND TREATMENT SUPPLY

Centers as well as the patients and health care providers involved in the care of the patients will know the treatment arm currently in place as the strategy (i.e. the two antibiotic regimen options and standard wound dressing versus Prevena in diabetic and obese patients) will be applied to all patients undergoing an eligible surgical procedure during

the study period. The antibiotics as well as the wound dressings used are approved and as such accessible to the investigators via routine standard of care.

5.4.3 DATA COLLECTION AND REPORTING

In order to maintain feasibility and reduce costs, we will limit data collection to the most essential data. We will use a secure online research data capture platform for data collection similar to what we are using in our current vanguard study.
Patients are routinely seen on a daily basis during their hospital stay. Once discharged or transferred to rehabilitation, patients would typically present in the surgical center if developing signs or symptoms of an infection. At most cardiac surgery centers, patients are routinely seen about 1 month after surgery. These points of contact will capture the vast majority of infections. In order to avoid duplication of work and to optimize the use of resources considering that Infection Prevention and Control (IPAC) programs usually conduct routine infection surveillance for s-SSIs, data will be collected by IPAC programs at each site whenever possible. Only if such a system is not yet in place and there is no interest in establish prospective surveillance by IPAC, the research team will collect their own data without support by the local IPAC program.
We have previously shown that routine surveillance data is as reliable as data collected by a dedicated research program if adding blinded outcome adjudication to a pre-existing routine surveillance program, and by having the IPAC program following up with the patients 90 days after surgery with a phone call if no follow-up visit at the site was conducted.[2] While the hourly wage of infection preventionists is higher than the wage of research assistants, infection preventionists are already collecting most of the information for surveillance purposes and are more likely to collect high-quality data given that this is the work they are doing routinely. In order to provide the needed additional resources to the IPAC programs for data collection, and if not yet standard of care at a site, the 3 months follow-up call, study sites can use (part of) the local budget to bolster their pre-existing surveillance program. In our current vanguard study, we were able to have full follow-up data for 96.9% of patients who were alive by calling patients three months post-op.

5.5 STUDY OUTCOME EVENTS

5.5.1 PROPOSED VANGUARD STUDY

The primary outcomes for the vanguard study are feasibility outcomes:

- Adherence to the wound management system as per protocol (goal >90%)
- Adherence to the antibiotic regimen as per protocol (goal >90%)
- Loss of follow-up (goal <10%)

DocuSign Envelope ID: 2F66E700-BE3D-413C-BA9A-47B956666541

In addition, data for the outcomes of the eventual full trial will be collected (see below).

5.5.2 EVENTUAL FULL TRIAL

5.5.2.1 PRIMARY OUTCOME

Composite outcome of both deep incisional and organ/space s-SSI following Center for Disease Control and Prevention/National Healthcare Safety Network (CDC/NHSN) definitions (Appendix A).[55]

5.5.2.2 SECONDARY OUTCOMES

1. All s-SSI including superficial incisional infections
2. SSI on the leg site (in the subgroup of patients with open venous saphenous harvest)
3. Wound dehiscence
4. Laboratory confirmed *C. difficile* infection
5. Mortality in patients with an active infection.
6. Length of ICU- and hospital stay
7. Pain at day 5 (+/- 1 day) using routinely collected Visual Analogue Scale (VAS) assessments
8. Acute kidney injury (AKI) within 7 days of the surgical procedure (based on serum creatinine, following Acute Kidney Injury Network definition[25])

5.5.3 OUTCOME ADJUDICATION IN THE EVENTUAL FULL TRIAL

We recognize the potential for bias related to patients and health care workers not being blinded. To mitigate against the possibility for differential outcome ascertainment as a result of lack of blinding, superficial s-SSI will not be considered for the primary outcome. This is due to subjectivity in making the diagnosis that may be more affected by the lack of blinding, along with their relatively minor clinical importance compared to deep incisional and organ/space s-SSI which have major implication on patients. Due to concerns about subjectivity and therefore bias, we will furthermore not define s-SSI based on a surgeon's assessment. We will nevertheless conduct a sensitivity analysis in which the surgeons' assessment (which will be collected) will be used to define s-SSIs. Most importantly, an outcome adjudication committee blinded to the study groups will assess the outcomes using standardized CDC/NHSN definitions based on presentation of the cases by infection preventionists or research personal.[55]

<u>Patient who are qualifying for a review by the outcome adjudication committee:</u> All patients with potential signs or symptoms of an infection that cannot be clearly attributed to an infectious focus other than the surgical site(s), and all patients with (suspected) SSI will be presented by the infection preventionist or study personal to the outcome adjudication committee. These signs and symptoms that trigger review by the committee

include all criteria listed in the CDC/NHSN definition (Appendix A) such as localized symptoms at the surgical site, discharge, dehiscence of the wound, whether the wound was deliberately opened by a surgeon, evidence of an abscess, microbiological samples if available, and the need for revision surgery, but also fever as a potential indicator of a s-SSI.
If the only symptom was either fever or pain/tenderness at the sternal surgical site with documentation of no signs of a sternal SSI at that time, these cases will not need to be presented and can be adjudicated as 'no SSI' by the infection preventionist/study personal. However, all patients with the lightest suspicion of a SSI will be presented to the outcome adjudication committee in a blinded manner. The same rules apply to patient with no telephone follow-up at 90 days if the documentation available is suspicious for a SSI.

Case reports: The infection control practioner/research personal at each site will draft a brief case report for cases meeting the requirement above. In order to prevent the outcome adjudication committee from guessing the study arm the patient was in, all dates must be replaced by related time periods, e.g. instead of reporting a date of the surgical procedure, and a date for onset of symptoms, the case report would report the post-op day when the symptoms occurred. Also, the study numbers must be replaced by an outcome adjudication number, and the reports must not be presented in chronological order. The code to cross reference the outcome adjudication number with the study number will be kept at the study site and not shared with anyone else. An example of a report of a (likely) superficial infection below:

Adjudication #: 3
Demographics: 69, male
Days in hospital: 5
Details during hospital stay:

- Uncomplicated course during hospital stay

90 day follow-up:

- patient reports localized pain/tenderness with superficial purulent discharge; reports sternal wound opened deliberately by surgeon
    - No microbiology swab sent
    - No record of follow-up/consultation with surgeon

Assessment by the outcome adjudication committee: The outcome adjudication committee consists of three members, with at least one member being a cardiac surgeon and one member being a specialist in infectious diseases and/or hospital epidemiology. Case reports will be forwarded to the outcome adjudication committee along with an Excel sheet that lists the potential outcomes of the assessment, i.e. no infection, superficial infection, deep and/or organ-space infection, not able to adjudicate and more information needed. The appropriate category can be checked off.
The responses of the three adjudicators will be collated, and the decisions of all cases with agreement between the three adjudicators can be put into the electronic data collection

DocuSign Envelope ID: 2F66E700-BE3D-413C-BA9A-47B956666541

system. For cases with disagreement, a follow-up phone call involving the local research staff as well as the three adjudicators will be organized to discuss the outstanding cases and to agree on a final assessment of the case.

5.6 SAMPLE SIZE AND STATISTICAL METHODS

5.6.1 SAMPLE SIZE VANGUARD STUDY

For the proposed vanguard study, two study centers with a total of roughly 4,000 patients combined will be enrolled. This will result in 95% confidence intervals around the feasibility outcomes of less than +/- 1%. While such narrow confidence intervals for feasibility outcomes would not necessarily be needed, we need to enrol at least two sites to gain the necessary insight into potential challenges with the feasibility of the study design for the full trial.

5.6.2 SAMPLE SIZE CALCULATION EVENTUAL FULL TRIAL

The rate of the proposed primary outcome, deep and organ/space s-SSI, will range from 1.5 to 2.5%. These estimates are based on surveillance data at sites interested in participating (ranging from 1.5-2%) and from the recent published literature.[33,56-59] Considering that post-discharge surveillance may account for up to 40% of infections[18], and considering a potential effect of dual antibiotic prophylaxis, our estimated event rate for the sample size calculation was set to 1.8%. This expected rate is used for the sample size calculation for the comparison of the two antibiotic prophylaxis regimens for which all enrolled patients will be considered. As only patients at higher risk for s-SSIs, i.e. patients with diabetes mellitus and/or BMI of 30 or greater, are eligible for the Prevena wound management system, only patients meeting these criteria in the Prevena arms will be compared to the patients meeting these same criteria in the standard dressing arms. We estimate that about 70% of all patients that will be enrolled are either diabetic or obese based on our current vanguard data. The rate of s-SSI for this at-risk group for infections is estimated to be 2.2% in the sample size calculation below based on published data showing higher s-SSI rates in this group.[33,49-54] Based on consensus opinion of the investigators and the steering committee, the minimally important clinical difference (MCID) was defined as an absolute difference in deep and organ/space s-SSI of 0.63% (+/- 35% rel. difference), an effect size that is within the range of what had been shown previously for vancomycin combination treatment and negative pressure wound management systems in less rigorous studies in the cardiac surgery population[16,34]. The Type 1 error for the first comparison, type of wound management strategy, is set to 0.035, and to 0.015 for the second comparison, the choice of the antibiotic regimen.

Using an approach suggested by Giraudeau and Donner[60], we first calculated the sample size without consideration of clustering or cross over. To account for the intra-class and inter-period correlation (ICC and IPC), we assumed that the IPC is 0.9 times the ICC. This is based on the not yet published PADIT study that compared different antibiotic regimens

for pacemake implantation. We chose 0.005 as a non-conservative estimate for the ICC. We then further increased the sample size to account for a 5% loss to follow-up and 5% 90-day infection-unrelated mortality. These estimates are conservative and are based on the current vanguard study where the combined proportion of death and other loss of follow-up is 7.4%. Assuming a true s-SSI rate for deep and organ space s-SSI of 2.2% for the first comparison (the wound management) and 1.8 % for second comparison (antibiotic regimen), 19 hospital sites with on average 500 patients undergoing cardiac surgery per arm for a total of 36,334 patients (i.e. 25,434 for patients with either known diabetes mellitus and/or obesity defined as a BMI of 30 or greater for the comparison of the two wound management strategies) will need to be enrolled to have 84% and 82% power to detect a relative reduction in s-SSI rates by 35% (0.77 and 0.63% absolute difference for the first and second comparison, respectively, from a total of 589 expected s-SSIs during the study period).

Assuming that no major changes to the protocol are needed based on the experience from the proposed vanguard study outlined in this protocol, the data from these 2 sites with roughly 4,000 patients will be rolled over into the eventual full trial, thus, an additional 17 sites with 30,334 patients will be needed to power the eventual full trial as outlined above.

5.6.3 Analysis Plan

For the proposed vanguard study, feasbility outcomes will be reported as proportions and 95% confidence intervals.

For the full trial, baseline characteristics will be presented by treatment group using count and percent for categorical data and means and standard deviations for continuous data. We will apply hierarchical modelling (generalized linear mixed model) for the primary analysis in order to adjust for cluster effects, stratified according to the factorial allocation in the intention-to-treat patient population meeting inclusion criteria (SAS Version 9.4 for UNIX). As the data collection needs to be as parsimonious as possible we will not adjust for potential confounders for which we will not be collecting data such as nutrition status, duration and technique of the surgical procedure, glucose control, co-moribidities (other than diabetes mellitus, obesity, chronic obstructive pulmonary disease, and peripheral vascular disease for which data is being collected). Given the large size of the study, the randomization process, and the fact that each site serves as its own control, these confounding factors are expected to be well balanced between the two groups and the potential impact of these confounders is negligible. This approach will also model centers as random effects and periods as fixed effects to account for the correlation within a center (ICC) and within a period (IPC), respectively. The association between the treatment group and the outcomes will be reported as odds ratios with a 95% confidence interval. The analysis for the wound management will be limited to the patients with either known diabetes mellitus and/or obesity defined as a BMI of 30 or greater. For leg incision site infections, the eligible study population will be limited to those qualifying for Prevena, i.e. meeting the criteria above plus having had an open saphenous vein harvesting proceudre.

The pre-defined, exploratory subgroup analysis are: the use of bilateral mammary artery, and dialysis either pre- or post-operatively. We are hypothesizing that there is a larger

DocuSign Envelope ID: 2F66E700-BE3D-413C-BA9A-47B956666541

treatment effect with both interventions in patients deemed to be at higher risk based on these criteria listed.

In an exploratory analysis, we will model BMI to identify the best possible cut-offs for the prevention of s-SSI with Prevena by fitting the model with dichotomized BMI above and below specific thresholds using sensitivity, specificity, C statistics (95% CI), and the closest-to-(0,1) criterion, which is the minimum distance to point (0,1) on each outcome's respective receiver operating characteristic (ROC) curve. The optimal BMI will be the one with the smallest minimum distance.[61,62]

6.0 ADMINISTRATIVE AND REGULATORY

6.1 ETHICS REVIEW

Obtaining individual consent for this cluster RCT would jeopardize one of the main advantages of this design, i.e. testing the comparative effectiveness of the interventions in a real-world setting rather than testing efficacy in a subgroup of highly selected patients. Obtaining individual patient consent would not allow assessing the intervention in important groups of patients such as emergent and urgent surgery patients. Research ethics board can approve a waiver for individual informed consent if the following criteria are met: 1) no more than minimal risk to participants, 2) the alteration to consent requirements is unlikely to adversely affect the welfare of participants, 3) it is impossible or impracticable to carry out the research properly given the research design if prior consent is needed, 4) there is a plan to offer participants the possibility of withdrawing data.[63] Therefore, a waiver of consent or an opt-out option for patients is considered appropriate in this minimal risk clinical effectiveness study[64]. The REBs at the current vanguard sites for the PICS study agreed that these criteria are met and waived the need for individual patient consent. Patients will be informed about the study during the telephone follow-up three months after surgery or earlier as requested by the local research ethic boards, e.g. by providing a letter during the hospital stay as done in the current vanguard study. A waiver of consent was also approved at all 28 sites (in Canada and the Netherlands) for the PADIT trial[1] (unpublished data).

6.2 GOOD CLINICAL PRACTICE (GCP)

The procedures set out in this protocol are designed to ensure that the investigator abide by the principles of the Declaration of Helsinki and Good Clinical Practice Guidelines (ICH-GCP) in the latest version, in conduct, evaluation and documentation of the study.

6.3 TIMELINES AND MILESTONES

Based on experience from our current vanguard study, it will take approximately 6 months from the initial agreement until a study site is able to start enrolment. Logistical issues around the dual arm (e.g. ensuring that the pumps for the administration of vancomycin

DocuSign Envelope ID: 2F66E700-BE3D-413C-BA9A-47B956666541

are available on time) as well as training of the appropriate use of the Prevena wound management system need to be planned well in advance of the study launch. We anticipate that the two proposed vanguard sites can launch the study in early 2018. Assuming that high-volume centers will be recruited for the vanguard study, we expect enrolment to be completed after 20 months (4 month duration of each arm, 1 month phase-in for each arm), followed by a three months follow-up period to ascertain outcomes.
For the eventual full trial, we anticipate that the start dates for the sites will be spread over 6 months, such that after 12 months we expect all sites to have begun to enroll patients. The duration of the study at each site will be 20-36 months, this will include four 4-8-month blocks for each of the 4 study arms along with four 1-month wash-in periods. We anticipate data collection to be complete 3 months after the last patient is enrolled. The outcome adjudication committee will complete the review of patients that were deemed to have possible infection. We estimate that this process will require another 2 months. Estimating another 3 months to complete data cleaning and analysis, we anticipate that the total duration of the study will be 48 months. These milestones will serve as performance measures that will be reported to the steering committee. Failure to meet these timelines will result in corrective action.
Recruitment of study sites will be facilitated by the fact that this study will build upon an existing CIHR research network (Canadian Network and Centre for Trials Internationally (CANNeCTIN; www.cannectin.ca)). Study sites that expressed interest in participating in the here proposed trial include Canadian centers as well as centers in Switzerland and the Netherlands. Furthermore, the study can benefit from the research collaboration of the perioperative research program at the Population Health Research Institute (PHRI).

Note: If due to an unforeseen circumstance (e.g. COVID-19) where surgeries may stop or be reduced, the study arm may be extended. The new timeline for the study arm will need approval by the study Principal Investigator.

6.4 DATA SAFETY AND MONITORING BOARD

An independent data safety monitoring board (DSMB) will be comprised of experts in the relevant clinical areas. The committee will review the study data in an unblinded manner and may request an interim analysis if deemed appropriate. If so, an alpha control method such as the O'Brien-Fleming group sequential boundaries with the Lan-DeMets alpha-spending function approach will be suggested to the DSMB.

6.5 STATEMENT OF CONFIDENTIALITY

All patient information will be de-identified using code numbers to correspond to treatment data. Data analysis will be performed on a high security computer system. Disclosure of data collected in the trial to third parties, is prohibited with the exceptions noted below.

DocuSign Envelope ID: 2F66E700-BE3D-413C-BA9A-47B956666541

6.6 AUDIT AND INSPECTION

The Investigator/Institution will permit study related monitoring, audits, REB and regulatory inspections, providing direct access to all related source data/documents by PHRl or Canadian health authorities (i.e. Health Canada), or other regulatory bodies.

We recognize that adherence to the study protocol needs buy-in from cardiac surgeons, anesthesiologists, operating room personal as well as from personal in pre-operative care, same day surgery units, wards and intensive care units. All these groups will be involved from the outset prior to the launch of the study, there will be ongoing feedback and reminders through emails, research boards etc., and at least 5% of randomly selected patient's health records at each site will be audited in the eventual full trial to ensure adherence to the allocated strategy. Adherence to the protocol will serve as a performance measure and will be reported to the data safety and monitoring board as well as to the steering committee. In the current vanguard study, adherence to the protocol has been above the 95% target: the pre-operative dose was not given according to the protocol in 3.1% of cases, the intra-operative dose in 2.7%, and the post-operative antibiotics in 2.2% of cases.

6.7 ROLE OF THE INDUSTRY SPONSOR

The research protocol was drafted by the academic investigators. The industry sponsor, Acelity, had the right to propose changes to the protocol which were considered by the academic investigators. The industry sponsor will not have a role in conducting the trial other than supporting education on their product at the study sites. Data collection and analysis will be conducted by the academic investigators and the study personal at the study sites, and the industry sponsor will not have access to the raw data. The industry sponsor has the right to review and provide feedback on abstracts and manuscripts before submission. All final decisions regarding publication and reporting will be made by the academic investigators.

7.0 SCIENTIFIC REPORTING AND PUBLICATION

The study protocol was developed by the Principal Investigator and Study Steering Committee. The Principal Investigator in collaboration with the Steering Committee is responsible for the scientific reporting, publishing and/or presentation of the study results. The information developed during the conduct of this clinical study is considered confidential.

We anticipate that the eventual full trial will be a landmark study which will define the choice of antimicrobial prophylaxis and wound management to prevent s-SSIs. Specifically, we will determine whether a dual regimen including vancomycin and a negative-pressure wound management technology (Prevena) are superior to cefazolin

DocuSign Envelope ID: 2F66E700-BE3D-413C-BA9A-47B956666541

single-agent prophylaxis and standard of care wound dressing, respectively. Our trial will either confirm or refute recommendations that specify that antibiotic prophylaxis with cefazolin mono-therapy should be routine practice[15], or it may support the practise of using a dual prophylaxis including vancomycin, a practise which is becoming routine in particularly in the U.S. Our findings will be presented at Population Health Research Institute (PHRI) symposia, international conferences of key stakeholders (surgery, anaesthesiology, infectious diseases), and published in an open access journal. Furthermore, the findings will be shared across the existing CIHR research network (Canadian Network and Centre for Trials Internationally (CANNeCTIN)) to allow a quicker translation of the findings into practice at key centers with which McMaster University and PHRI are collaborating with.

8.0 REFERENCES

1. Connolly SJ, Philippon F, Longtin Y, et al. Randomized cluster crossover trials for reliable, efficient, comparative effectiveness testing: design of the Prevention of Arrhythmia Device Infection Trial (PADIT). *Can J Cardiol.* 2013;29(6):652-658.
2. Mertz D, Whitlock R, Kokoszka AY, et al. Routine Surveillance Versus Independent Assessment by an Outcome Adjudication Committee in Assessing Patients for Sternal Surgical Site Infections After Cardiac Surgery. *Infection control and hospital epidemiology.* 2016:1-3.
3. Filsoufi F, Castillo JG, Rahmanian PB, et al. Epidemiology of deep sternal wound infection in cardiac surgery. *Journal of Cardiothoracic and Vascular Anesthesia.* 2009;23(4):488-494.
4. Hillis LD, Smith PK, Anderson JL, et al. 2011 ACCF/AHA Guideline for Coronary Artery Bypass Graft Surgery: executive summary: a report of the American College of Cardiology Foundation/American Heart Association Task Force on Practice Guidelines. *Circulation.* 2011;124(23):2610-2642.
5. Graf K, Ott E, Vonberg RP, Kuehn C, Haverich A, Chaberny IF. Economic aspects of deep sternal wound infections. *European journal of cardio-thoracic surgery : official journal of the European Association for Cardio-thoracic Surgery.* 2010;37(4):893-896.
6. Edwards FH, Engelman RM, Houck P, Shahian DM, Bridges CR. The Society of Thoracic Surgeons Practice Guideline Series: Antibiotic Prophylaxis in Cardiac Surgery, Part I: Duration. *Ann Thorac Surg.* 2006;81(1):397-404.
7. Graf K, Ott E, Vonberg RP, Kuehn C, Haverich A, Chaberny IF. Economic aspects of deep sternal wound infections. *Eur J Cardiothorac Surg.* 2009.
8. Taylor GJ, Mikell FL, Moses HW, et al. Determinants of hospital charges for coronary artery bypass surgery: the economic consequences of postoperative complications. *Am J Cardiol.* 1990;65(5):309-313.

DocuSign Envelope ID: 2F66E700-BE3D-413C-BA9A-47B956666541

9. Lador A, Nasir H, Mansur N, et al. Antibiotic prophylaxis in cardiac surgery: systematic review and meta-analysis. *The Journal of antimicrobial chemotherapy*. 2012;67(3):541-550.
10. Mertz D, Johnstone J, Loeb M. Does duration of perioperative antibiotic prophylaxis matter in cardiac surgery? A systematic review and meta-analysis. *Annals of Surgery*. 2011;254(1):48-54.
11. Hsiue PP, Gregson AL, Injean P, et al. Variation in Antibiotic Prophylaxis Selection for Coronary Artery Bypass Graft Procedures in an Era of Increasing Methicillin-Resistant Staphylococcus aureus Prevalence. *Infection control and hospital epidemiology : the official journal of the Society of Hospital Epidemiologists of America.* 2014;35(6):737-740.
12. Poeran J, Mazumdar M, Rasul R, et al. Antibiotic prophylaxis and risk of Clostridium difficile infection after coronary artery bypass graft surgery. *The Journal of thoracic and cardiovascular surgery*. 2015.
13. Gorski A, Hamouda K, Ozkur M, et al. Cardiac surgery antibiotic prophylaxis and calculated empiric antibiotic therapy. *Asian Cardiovasc Thorac Ann.* 2015;23(3):282-288.
14. Engelman R, Shahian D, Shemin R, et al. The Society of Thoracic Surgeons practice guideline series: Antibiotic prophylaxis in cardiac surgery, part II: Antibiotic choice. *Ann Thorac Surg.* 2007;83(4):1569-1576.
15. Bratzler DW, Dellinger EP, Olsen KM, et al. Clinical practice guidelines for antimicrobial prophylaxis in surgery. *American journal of health-system pharmacy : AJHP : official journal of the American Society of Health-System Pharmacists.* 2013;70(3):195-283.
16. Branch-Elliman W, Ripollone JE, O'Brien WJ, et al. Risk of surgical site infection, acute kidney injury, and Clostridium difficile infection following antibiotic prophylaxis with vancomycin plus a beta-lactam versus either drug alone: A national propensity-score-adjusted retrospective cohort study. *PLoS Med.* 2017;14(7):e1002340.
17. Nationales Referenzzentrum fuer Surveillance von nosokomialen Infektionen. Modul OP-KISS (Surveillance System postoperative wound infections). www.nrz-hygiene.de. Accessed February 19, 2013.
18. Cossin S, Malavaud S, Jarno P, et al. Surgical site infection after valvular or coronary artery bypass surgery: 2008-2011 French SSI national ISO-RAISIN surveillance. *The Journal of hospital infection.* 2015;91(3):225-230.
19. Yavuz SS, Tarcin O, Ada S, et al. Incidence, aetiology, and control of sternal surgical site infections. *The Journal of hospital infection.* 2013;85(3):206-212.
20. Walsh EE, Greene L, Kirshner R. Sustained reduction in methicillin-resistant Staphylococcus aureus wound infections after cardiothoracic surgery. *Arch Intern Med.* 2011;171(1):68-73.
21. Garey KW, Lai D, Dao-Tran TK, Gentry LO, Hwang LY, Davis BR. Interrupted time series analysis of vancomycin compared to cefuroxime for surgical prophylaxis in patients undergoing cardiac surgery. *Antimicrob Agents Chemother.* 2008;52(2):446-451.

DocuSign Envelope ID: 2F66E700-BE3D-413C-BA9A-47B956666541

22. White RW, West R, Howard P, Sandoe J. Antimicrobial regime for cardiac surgery: the safety and effectiveness of short-course flucloxacillin (or teicoplanin) and gentamicin-based prophylaxis. *J Card Surg.* 2013;28(5):512-516.
23. Engemann JJ, Carmeli Y, Cosgrove SE, et al. Adverse clinical and economic outcomes attributable to methicillin resistance among patients with Staphylococcus aureus surgical site infection. *Clinical infectious diseases : an official publication of the Infectious Diseases Society of America.* 2003;36(5):592-598.
24. Finkelstein R, Rabino G, Mashiah T, et al. Vancomycin versus cefazolin prophylaxis for cardiac surgery in the setting of a high prevalence of methicillin-resistant staphylococcal infections. *The Journal of thoracic and cardiovascular surgery.* 2002;123(2):326-332.
25. Acute Kidney Injury Network. 2016; http://www.akinet.org/akinstudies.php. Accessed Nov 6, 2017.
26. Schweizer M, Perencevich E, McDanel J, et al. Effectiveness of a bundled intervention of decolonization and prophylaxis to decrease Gram positive surgical site infections after cardiac or orthopedic surgery: systematic review and meta-analysis. *BMJ.* 2013;346:f2743.
27. Morykwas MJ, Argenta LC, Shelton-Brown EI, McGuirt W. Vacuum-assisted closure: a new method for wound control and treatment: animal studies and basic foundation. *Annals of plastic surgery.* 1997;38(6):553-562.
28. Mendonca DA, Papini R, Price PE. Negative-pressure wound therapy: a snapshot of the evidence. *Int Wound J.* 2006;3(4):261-271.
29. Grauhan O, Navasardyan A, Hofmann M, Muller P, Stein J, Hetzer R. Prevention of poststernotomy wound infections in obese patients by negative pressure wound therapy. *The Journal of thoracic and cardiovascular surgery.* 2013;145(5):1387-1392.
30. Stannard JP, Volgas DA, McGwin G, 3rd, et al. Incisional negative pressure wound therapy after high-risk lower extremity fractures. *J Orthop Trauma.* 2012;26(1):37-42.
31. Karlakki S, Brem M, Giannini S, Khanduja V, Stannard J, Martin R. Negative pressure wound therapy for managementof the surgical incision in orthopaedic surgery: A review of evidence and mechanisms for an emerging indication. *Bone Joint Res.* 2013;2(12):276-284.
32. Stannard JP, Volgas DA, Stewart R, McGwin G, Jr., Alonso JE. Negative pressure wound therapy after severe open fractures: a prospective randomized study. *J Orthop Trauma.* 2009;23(8):552-557.
33. Willy C, Engelhardt M, Stichling M, Grauhan O. The impact of surgical site occurrences and the role of closed incision negative pressure therapy. *Int Wound J.* 2016;13 Suppl 3:35-46.
34. Allegranzi B, Zayed B, Bischoff P, et al. New WHO recommendations on intraoperative and postoperative measures for surgical site infection prevention: an evidence-based global perspective. *Lancet Infect Dis.* 2016;16(12):e288-e303.

DocuSign Envelope ID: 2F66E700-BE3D-413C-BA9A-47B956666541

35. Sox HC, Lewis RJ. Pragmatic Trials: Practical Answers to "Real World" Questions. *Jama.* 2016;316(11):1205-1206.
36. Leon AC, Davis LL, Kraemer HC. The role and interpretation of pilot studies in clinical research. *J Psychiatr Res.* 2011;45(5):626-629.
37. Thabane L, Ma J, Chu R, et al. A tutorial on pilot studies: the what, why and how. *BMC Med Res Methodol.* 2010;10:1.
38. ASHP Therapeutic Guidelines on Antimicrobial Prophylaxis in Surgery. American Society of Health-System Pharmacists. *Am J Health Syst Pharm.* 1999;56(18):1839-1888.
39. Bratzler DW, Houck PM. Antimicrobial prophylaxis for surgery: an advisory statement from the National Surgical Infection Prevention Project. *Clin Infect Dis.* 2004;38(12):1706-1715.
40. Eagle KA, Guyton RA, Davidoff R, et al. ACC/AHA 2004 guideline update for coronary artery bypass graft surgery: summary article. A report of the American College of Cardiology/American Heart Association Task Force on Practice Guidelines (Committee to Update the 1999 Guidelines for Coronary Artery Bypass Graft Surgery). *Journal of the American College of Cardiology.* 2004;44(5):e213-310.
41. Gilbert DN, Moellering Jr. RC, Eliopoulos GM, Chambers HF, Saag MS. *The Sanford Guide To Antimicrobial Therapy.* 41st Edition ed2011.
42. Harbarth S, Samore MH, Lichtenberg D, Carmeli Y. Prolonged antibiotic prophylaxis after cardiovascular surgery and its effect on surgical site infections and antimicrobial resistance. *Circulation.* 2000;101(25):2916-2921.
43. Alvarez P, Fuentes C, Garcia N, Modesto V. Evaluation of the duration of the antibiotic prophylaxis in paediatric postoperative heart surgery patients. *Pediatr Cardiol.* 2012;33(5):735-738.
44. Colli A, Camara ML. First experience with a new negative pressure incision management system on surgical incisions after cardiac surgery in high risk patients. *J Cardiothorac Surg.* 2011;6:160.
45. Lee AJ, Sheppard CE, Kent WD, Mewhort H, Sikdar KC, Fedak PW. Safety and efficacy of prophylactic negative pressure wound therapy following open saphenous vein harvest in cardiac surgery: a feasibility study. *Interactive cardiovascular and thoracic surgery.* 2017;24(3):324-328.
46. Cheng D, Allen K, Cohn W, et al. Endoscopic vascular harvest in coronary artery bypass grafting surgery: a meta-analysis of randomized trials and controlled trials. *Innovations (Phila).* 2005;1(2):61-74.
47. Tennyson C, Young CP, Scarci M. Is it safe to perform endoscopic vein harvest? *Interactive cardiovascular and thoracic surgery.* 2010;10(4):625-629.
48. Reed JF, 3rd. Leg wound infections following greater saphenous vein harvesting: minimally invasive vein harvesting versus conventional vein harvesting. *Int J Low Extrem Wounds.* 2008;7(4):210-219.
49. Figuerola-Tejerina A, Rodriguez-Caravaca G, Bustamante-Munguira J, Maria San Roman-Montero J, Duran-Poveda M. Epidemiological Surveillance of

DocuSign Envelope ID: 2F66E700-BE3D-413C-BA9A-47B956666541

Surgical Site Infection and its Risk Factors in Cardiac Surgery: A Prospective Cohort Study. *Rev Esp Cardiol.* 2016;69(9):842-848.
50. Ledur P, Almeida L, Pellanda LC, Schaan BD. Predictors of infection in post-coronary artery bypass graft surgery. *Rev Bras Cir Cardiovasc.* 2011;26(2):190-196.
51. Bellchambers J, Harris JM, Cullinan P, Gaya H, Pepper JR. A prospective study of wound infection in coronary artery surgery. *European journal of cardio-thoracic surgery : official journal of the European Association for Cardio-thoracic Surgery.* 1999;15(1):45-50.
52. Salehi Omran A, Karimi A, Ahmadi SH, et al. Superficial and deep sternal wound infection after more than 9000 coronary artery bypass graft (CABG): incidence, risk factors and mortality. *BMC Infect Dis.* 2007;7:112.
53. Schimmer C, Gross J, Ramm E, et al. Prevention of surgical site sternal infections in cardiac surgery: a two-centre prospective randomized controlled study. *European journal of cardio-thoracic surgery : official journal of the European Association for Cardio-thoracic Surgery.* 2016.
54. Colombier S, Kessler U, Ferrari E, von Segesser LK, Berdajs DA. Influence of deep sternal wound infection on long-term survival after cardiac surgery. *Med Sci Monit.* 2013;19:668-673.
55. Center for Disease Control and Prevention/National Healthcare Safety Network (CDC/NHSN): Surveillance Definitions for Specific Types of Infections. http://www.cdc.gov/nhsn/PDFs/pscManual/9pscSSIcurrent.pdf?agree=yes&next=Accept (accessed November 20, 2015).
56. Austin TW, Coles JC, Burnett R, Goldbach M. Aortocoronary bypass procedures and sternotomy infections: A study of antistaphylococcal prophylaxis. *Canadian Journal of Surgery.* 1980;23(5):483-485.
57. Gupta A, Hote MP, Choudhury M, Kapil A, Bisoi AK. Comparison of 48 h and 72 h of prophylactic antibiotic therapy in adult cardiac surgery: a randomized double blind controlled trial. *The Journal of antimicrobial chemotherapy.* 2010;65(5):1036-1041.
58. Saginur R, Croteau D, Bergeron MG. Comparative efficacy of teicoplanin and cefazolin for cardiac operation prophylaxis in 3027 patients. *Journal of Thoracic and Cardiovascular Surgery.* 2000;120(6):1120-1130.
59. Frenette C, Sperlea D, Tesolin J, Patterson C, Thirion DJ. Influence of a 5-year serial infection control and antibiotic stewardship intervention on cardiac surgical site infections. *Am J Infect Control.* 2016;44(9):977-982.
60. Giraudeau B, Ravaud P, Donner A. Sample size calculation for cluster randomized cross-over trials. *Stat Med.* 2008;27(27):5578-5585.
61. Liu X. Classification accuracy and cut point selection. *Stat Med.* 2012;31(23):2676-2686.
62. Yazdan-Ashoori P, Lee SF, Ibrahim Q, Van Spall HG. Utility of the LACE index at the bedside in predicting 30-day readmission or death in patients hospitalized with heart failure. *American heart journal.* 2016;179:51-58.

DocuSign Envelope ID: 2F66E700-BE3D-413C-BA9A-47B956666541

63. Tri-Council Policy Statement. Ethical Conduct for Research Involving Humans. 2014. http://www.pre.ethics.gc.ca/pdf/eng/tcps2-2014/TCPS_2_FINAL_Web.pdf, Accessed February 2 2016.
64. McKinney RE, Jr., Beskow LM, Ford DE, et al. Use of altered informed consent in pragmatic clinical research. *Clin Trials.* 2015;12(5):494-502.

APPENDIX A

Definition of sternal surgical site infections according to Center for Disease Control and Prevention/National Healthcare Safety Network (CDC/NHSN) Surveillance Definitions for Specific Types of Infections from:
(http://www.cdc.gov/nhsn/PDFs/pscManual/9pscSSIcurrent.pdf?agree=yes&next=Accept)

Deep incisional SSI
Must meet the following criteria:
Infection occurs within 30 or 90 days after the NHSN operative procedure (where day 1 = the procedure date) according to the list in Table 2
AND
involves deep soft tissues of the incision (e.g., fascial and muscle layers)
AND
patient has at least ***one*** of the following:

a. purulent drainage from the deep incision.
b. a deep incision that spontaneously dehisces, or is deliberately opened or aspirated by a surgeon, attending physician** or other designee and organism is identified by a culture or non-culture based microbiologic testing method which is performed for purposes of clinical diagnosis or treatment (e.g., not Active Surveillance Culture/Testing (ASC/AST) or culture or non-culture based microbiologic testing method is not performed
   AND
   patient has at least ***one*** of the following signs or symptoms: fever (>38°C); localized pain or tenderness. A culture or non-culture based test that has a negative finding does not meet this criterion.
c. an abscess or other evidence of infection involving the deep incision that is detected on gross anatomical or histopathologic exam, or imaging test

** The term attending physician for the purposes of application of the NHSN SSI criteria may be interpreted to mean the surgeon(s), infectious disease, other physician on the case, emergency physician or physician's designee (nurse practitioner or physician's assistant).

Organ/Space SSI
Must meet the following criteria:

Infection occurs within 30 or 90 days after the NHSN operative procedure (where day 1 = the procedure date) according to the list in Table 2

DocuSign Envelope ID: 2F66E700-BE3D-413C-BA9A-47B956666541

AND
infection involves any part of the body deeper than the fascial/muscle layers, that is opened or manipulated during the operative procedure
AND
patient has at least ***one*** of the following:

a. purulent drainage from a drain that is placed into the organ/space (e.g., closed suction drainage system, open drain, T-tube drain, CT guided drainage)
b. organisms are identified from an aseptically-obtained fluid or tissue in the organ/space by a culture or non-culture based microbiologic testing method which is performed for purposes of clinical diagnosis or treatment (e.g., not Active Surveillance Culture/Testing (ASC/AST).
c. an abscess or other evidence of infection involving the organ/space that is detected on gross anatomical or histopathologic exam, or imaging test

AND
meets at least ***one*** criterion for a specific organ/space infection site listed in Table 3. These criteria are found in the Surveillance Definitions for Specific Types of Infections chapter.

DocuSign Envelope ID: 2F66E700-BE3D-413C-BA9A-47B956666541

MED-Mediastinitis

Mediastinitis must meet at least ***one*** of the following criteria:

1. Patient has organisms identified from mediastinal tissue or fluid by a culture or non-culture based microbiologic testing method which is performed for purposes of clinical diagnosis or treatment (e.g., not Active Surveillance Culture/Testing (ASC/AST).
2. Patient has evidence of mediastinitis on gross anatomic or histopathologic exam.
3. Patient has at least ***one*** of the following signs or symptoms: fever (>38.0°C), chest pain*, or sternal instability*
   And at least *one* of the following:
   a. purulent drainage from mediastinal area
   b. mediastinal widening on imaging test
4. Patient ≤1 year of age has at least ***one*** of the following signs or symptoms: fever (>38.0°C), hypothermia (<36.0°C), apnea*, bradycardia*, or sternal instability*
   And at least *one* of the following:
   a. purulent drainage from mediastinal area
   b. mediastinal widening on imaging test

** With no other recognized cause*

Reporting instruction

- Mediastinal space is the area under the sternum and in front of the vertebral column, containing the heart and its large vessels, trachea, esophagus, thymus, lymph nodes, and other structures and tissues. It is divided into anterior, middle, posterior, and superior regions.
- Report mediastinitis (MED) following cardiac surgery that is accompanied by osteomyelitis as SSI-MED rather than SSI-BONE.
