# Supplementary material for: Prevention of Infections in Cardiac Surgery (PICS)-Prevena Study – A pilot/vanguard factorial cluster cross-over RCT
Source: PLoS One. 2025 Dec 15;20(12):e0338300. doi: 10.1371/journal.pone.0338300 (PMC12704892; doi:10.1371/journal.pone.0338300)
Supplement: S3 Appendix — (PDF) [file pone.0338300.s006.pdf]

Protocol No. / Trial: Version 4.0, dated 2022-08-08

Project: PREVENTION OF INFECTIONS IN CARDIAC SURGERY (PICS) PREVENA VANGUARD STUDY:

A CLUSTER-RANDOMIZED FACTORIAL CROSSOVER TRIAL, COMPARING ANTIBIOTIC MONO-PROPHYLAXIS WITH CEFAZOLIN VS. DUAL-PROPHYLAXIS WITH CEFAZOLIN PLUS VANCOMYCIN AND CONVENTIONAL WOUND DRESSING VS. PREVENA NEGATIVE-PRESSURE WOUND MANAGEMENT

STATISTICAL ANALYSIS PLAN

Lead SAP Author/Principal Investigator: Dominik Mertz
Study Statistician: Chinthanie Ramasundarahettige

Version 1.0

Date 2023-10-30

TABLE OF CONTENTS

LIST OF ABBREVIATIONS 3
1. INTRODUCTION 4
2. STUDY DESIGN 5
3. STUDY HYPOTHESES/OBJECTIVES 5
    Feasibility outcomes 5
    Eventual Full Trial 6
    Primary Outcome 7
    Secondary Outcomes 7
4. POPULATIONS TO BE ANALYZED 7
5. BASELINE CHARACTERISTICS 7
6. STUDY FOLLOW-UP TIME 7
7. ANALYSIS 8
    Other Exploratory Analysis 9
8. SAFETY ANALYSIS 9
    Serious or Serious Unanticipated Adverse Device Effects 9
9. SUBGROUP ANALYSIS 9
APPROVAL 10

LIST OF ABBREVIATIONS

BMI: Body Mass Index
CANNeCTIN: Canadian Network and Centre for Trials Internationally
DSMB: Data Safety Monitoring Board
FDA: Food and Drug Administration
ICC: Intra-class Correlation
IPC: Inter-period Correlation
ICU: Intensive Care Unit
IPAC: Infection Prevention and Control
MRSA: Methicillin-resistant S. aureus
NHSN/CDC: National Healthcare Safety Network/Centres for Disease Control
OR: Operating Room
PADIT: Prevention of Arrhythmia Device Infection Trial
PHRI: Population Health Research Institute
PIMS: Prevena Incision Management System
RCT: Randomized Controlled Trials
s-SSI: Sternal Surgical Site Infection
VAS: Visual Analog Scale
WHO: World Health Organization

1. INTRODUCTION

The statistical analysis plan (SAP) specifies the details of the statistical analysis of the PICS-Prevena trial described in the Clinical Study Protocol (version 4.0, dated 2022-08-08). The SAP is a working document that will be amended as new information becomes available. Approval is provided for the content of the appendices at the time of approval. Appendices may be updated as required during the course of the study without obtaining approval for the changes; however, the author will inform those approving this document of updates to the appendices. The final version of the SAP will be signed off prior to database lock.

Despite the routine use of antibiotics before and after cardiac surgery, infections of the chest wound (sternal surgical site infection, s-SSI) remain a common life-threatening complication of heart surgery. Antibiotic prophylaxis is well accepted as the cornerstone of prevention for these infections; the best choice of antibiotic prophylaxis in patients undergoing open heart surgery is, however, unclear. Roughly 40% of pathogens identified in chest wound infections, such as coagulase-negative staphylococci and methicillin-resistant S. aureus (MRSA), are not covered by the recommended cefazolin prophylaxis, hence, there is large variability in practice. On the other hand, the risks associated with unnecessary use of antibiotics are well documented and include the emergence of "superbugs" and Clostridium difficile infections. Furthermore, negative pressure wound management systems can reduce the risk of SSIs by sealing the surgical site, removing potentially infected exudates, and improving wound healing. This Health Canada approved technology is showing promising results in small and non-randomized studies in cardiac surgery as well as other disciplines, but there is a lack of high-level evidence to support its use.

The proposed vanguard with 2 sites of the eventual multi-centre 2x2 factorial cluster randomized crossover trial with 18 sites was designed to test whether dual antibiotic prophylaxis with cefazolin plus vancomycin is superior to cefazolin mono-prophylaxis, and whether a negative-pressure wound management system (Prevena) is superior to standard wound dressing.

Due to COVID-19 surgeries across centres had stopped or had been reduced. Protocol Version 2.0 indicated each arm will be 4-8 months. We had to amend the protocol to allow the study arm to be extended for these unforeseen circumstances. We had 3 temporary stoppages from our site in London, Ontario:

1. June 1, 2020 until November 1, 2020 due to COVID-19 and lack of study staff.
2. November 17, 2021 until April 13, 2022 because of no resupply of Prevena Negative Wound Dressings because the company was making other COVID-19 products.
3. July 30, 2022 until October 31, 2022 because of the protocol amendment to increase total recruitment number at this site.

2. STUDY DESIGN

This study is a multi-center, 2x2 factorial, cluster crossover study with 4 periods. Centers were randomized to one of eight orders of the four study arms:

1) cefazolin prophylaxis plus Prevena,
2) cefazolin and vancomycin prophylaxis plus Prevena,
3) cefazolin prophylaxis plus standard wound dressing,
4) cefazolin and vancomycin prophylaxis plus standard wound dressing.

Each study arm was implemented for 4-8 months, aiming for approximately 500 patients per period per site. One of the four arms, randomized to each site-period, became the standard of care for all patients undergoing cardiac surgery at the site during that time-period. A wash-in period of one month prior to each arm was implemented to allow for the transition in management strategies. Data was not collected during the wash-in period.

Prevena was only made available to high-risk patients with diabetic or obese patients (BMI >30kg/m^2).

3. STUDY HYPOTHESES/OBJECTIVES

The objectives of this SAP for the vanguard study, which involves 2 sites completing four periods, are as follows.

1. To evaluate the feasibility of the study design.

2. To determine the effects of a negative-pressure wound management system (Prevena) on sternal surgical site infections in high-risk patients (i.e. diabetes or BMI>30kg/m^2) undergoing cardiac surgery.

Feasibility outcomes

The primary outcomes for the vanguard study are feasibility outcomes:

1. Adherence to the wound management system as per protocol (goal >90%) for high-risk patients undergoing cardiac surgery.

   There are two types of the wound management system.

- Adherence for the sternal wound
- Adherence for leg incision site in the eligible study population (the criteria above plus having had an open saphenous vein harvesting procedure).

Wound management system information is collected on the Surgical Visit CRF 2 Question A. Intervention 1. Cefazolin plus Prevena and Cefazolin and vancomycin plus Prevena → secondary question "Was Prevena used?" Yes/No and Type Sternal/Leg.

2. Adherence to the antibiotic regimen as per protocol (goal >90%) for selected 6 % of patients undergoing cardiac surgery.

Adherence to the antibiotic regimen will be summarized by treatment group (Cefazolin and Vancomycin vs Cefazolin, Prevena vs Standard for high risk patients only, each of the four study arm, during pre-op, intra-op and post-op. Unclear, which is "antibiotic ordered by no documented administration", is considered as adhered to given observations at the study sites that suggest that this was first and foremost a documentation issue with the old health information system. A successful trial will be defined by an overall adherence rate exceeding 90%.

3. Loss of follow-up (goal <10%) for both high-risk and all patients undergoing cardiac surgery.

We had given the participant to opt-out of the study and to remove all data by the site completing CRF 100. At the 90-day visit, we have asked if the visit was completed at Question 2. If not, then the site would need to specify the reason.

Patient refused further contact	*01*
No hospital or office visit information and unable to contact patient	*02*

Code 01 should not be considered as a lost to follow-up participant.
Code 02 should be considered as a lost to follow-up participant.

Loss of follow-up is defined as if the participant to opt-out of the study or if the 90-day visit was not completed and the reason is either "Patient refused further contact" or "No hospital or office visit information and unable to contact patient".

Eventual Full Trial

Since we have not secured funding for the full trial yet, we will report the primary and secondary outcomes for all patients without comparing the effectiveness of the antibiotic regimens.

Given that the industry sponsor for the wound management system will not continue to fund the full trial but is interested in the results, we will analyze these outcomes for high-risk patients eligible for the Prevena dressing, comparing the surgical site infection rates as a function of allocated wound management systems (i.e., Prevena vs. Standard wound dressing).

Primary Outcome

Composite outcome of both deep incisional and organ/space s-SSI following Center for Disease Control and Prevention/National Healthcare Safety Network (CDC/NHSN) definitions.

Secondary Outcomes

1. All s-SSI including superficial incisional infections
2. SSI on the leg site (in the subgroup of patients with open venous saphenous harvest)
3. Wound dehiscence
4. Laboratory confirmed C. difficile infection
5. Mortality in patients with an active infection.
6. Length of ICU- and hospital stay
7. Pain at day 5 (+/- 1 day) using routinely collected Visual Analogue Scale (VAS) assessments
8. Acute kidney injury (AKI) within 7 days of the surgical procedure (based on serum creatinine, following Acute Kidney Injury Network definition25)

4. POPULATIONS TO BE ANALYZED

We will follow to the intent-to-treat principle (ITT), which includes all patients admitted to the site undergoing cardiac surgery during that time period and randomized to one of the following study arms (as per Surgical Visit CRF 2 Question A.1). The wound management arm (Prevena (1+2) vs Standard wound dressing(3+4) will be compared among high-risk patients with diabetic or obese patients (BMI >30kg/m^2).

The four study arms are:

1) cefazolin prophylaxis plus Prevena
2) cefazolin and vancomycin prophylaxis plus Prevena,
3) cefazolin prophylaxis plus standard wound dressing,
4) cefazolin and vancomycin prophylaxis plus standard wound dressing.

5. BASELINE CHARACTERISTICS

Baseline characteristics (Surgical Visit CRF 2 Question C1 through 4) will be presented for all patients as aggregated and for the high-patients as aggregated and by treatment group – Prevena vs Standard, using count and percent for categorical data and means and standard deviations for continuous data.

6. STUDY FOLLOW-UP TIME

All efforts were made to collect complete data for all participants in this study.

In general, missing values within follow up will be treated as 'missing'. Patients with missing 3-months data would be considered as having no infection for the primary outcome as we would expect a patient with the primary outcome to have had contact with their surgeon and typically having been re-admitted. No attempt will be made to impute any other missing post-randomization values and only observed values will be used for analysis.

We collected data at the surgical visit, discharge visit and the 90-day visit. If the site reported that the participant died at a visit, then they didn't have to complete the next scheduled visit.

Baseline (Surgery), Time Windows and Calculated Visits

The date of surgery (Day 1) found on CRF 2 is the reference for all time-related analyses. It is expected that all participants should complete a 90-day visit unless they had died earlier. Follow-up time will be defined as the date of last contact for an individual, or the death date if available.

7. ANALYSIS

The feasibility outcomes of the vanguard study will be summarized by treatment groups as proportions and proportions difference with 95% confidence interval.

For the primary and secondary eventual full trial outcomes, we will report the aggregated data for all patients undergoing cardiac surgery without comparing between the antibiotics regimens. Given that the industry sponsor for the wound management system will not continue to fund the full trial but is interested in the results, the eventual full trial outcomes will be analyzed comparing the wound management system (Prevena vs Standard).

We will use the hierarchical modelling (generalized logistic mixed model) for the primary eventual full trial outcome in the intention-to-treat high-risk patient population with diabetes mellitus or obesity BMI>30kg/m^2. Due to a small number of sites included in the vanguard study, the models will include centers as random effects to account for the correlation within a center (ICC) and adjust for the factorial allocation as a fixed effect. As a sensitivity analysis, we will assess the heterogeneity of the treatment effect across centers by examining the interaction between treatment and centers.

All the binary secondary eventual full trial outcome will be analyzed with the same approach as the primary eventual full trial outcome. All the continuous secondary eventual full trial outcomes will have the same adjustment but analyzed using the generalized linear mixed model.

The association between the treatment group will be reported as odds ratios with a 95% CI for binary outcomes and mean difference with a 95% CI for continuous outcomes.

Other Exploratory Analysis

In an exploratory analysis, we will model BMI to identify the best possible cut-offs for the prevention of s-SSI with all patients with dichotomized BMI above and below specific thresholds (28, 30, 32, 34, 36, 38, 40) using sensitivity, specificity, C statistics (95% CI), and the closest-to-(0,1) criterion, which is the minimum distance to point (0,1) on each outcome's respective receiver operating characteristic (ROC) curve. The optimal BMI cut-off will be the one with the smallest minimum distance.

8. SAFETY ANALYSIS

Serious or Serious Unanticipated Adverse Device Effects

We used one of the approved and marketed negative-pressure wound management systems, the PREVENA Incision Management System (PIMS). This system is being used in many surgical disciplines and has been used in studies in cardiac surgery at the sternal as well as the saphenous vein harvest site in the past. The product is FDA and Health Canada approved for use on surgical sites.

The SAE collection/reporting period began upon the start of each study arm that includes the Prevena device and for only those participants who received the Prevena product. This also included the Prevena phase-in week. SAE reporting started upon application of the device (surgery date) until it was removed from the participant (if removal date unknown then assessed up to 7 days from date of application (surgery date)).

The SAE description will include the nature of the experience (SAE term), the start date, the end date, the severity of each sign or symptom, the seriousness of the event or experience, relationship to study treatment, the course of action taken, outcome of the experience and device information.

9. SUBGROUP ANALYSIS

The pre-defined, exploratory subgroup analysis includes the use of bilateral mammary artery, and dialysis either pre- or post-operatively. We are hypothesizing that there is a larger treatment effect with both interventions in patients deemed to be at higher risk based on these criteria listed.

APPROVAL

Version #	1.0
Version Date	2023-10-30

By signing the below, I designate my approval of the above-named version of the PICS-Prevena Statistical Analysis Plan on behalf of all named authors.

**Name**	**Dr. Dominik Mertz**
**Role**	**Principal Investigator**
**Signature**	
**Date** (yyyy/mm/dd)	

By signing the below, I designate my approval of the above-named version of the PICS-Prevena Statistical Analysis Plan on behalf of PHRI Statistics.

**Name**	**Chinthanie Ramasundarahettige**
**Role**	**Study Statistician**
**Signature**	
**Date** (yyyy/mm/dd)	
