# Supplementary material for: Prevention of Infections in Cardiac Surgery (PICS)-Prevena Study – A pilot/vanguard factorial cluster cross-over RCT
Source: PLoS One. 2025 Dec 15;20(12):e0338300. doi: 10.1371/journal.pone.0338300 (PMC12704892; doi:10.1371/journal.pone.0338300)
Supplement: S4 Appendix — (PDF) [file pone.0338300.s007.pdf]

Supplementary Material

Table of Contents

1 STUDY GROUPS 2

1.1 Project Office Operations Committee 2

1.2 Steering Committee 2

1.3 Data and Safety Monitoring Board (DSMB) 2

1.4 Adjudicators 2

1.5 Project Office Staff 2

1.6 Study Statisticians and Programmers 2

1.7 Participating Centres 2

1 STUDY GROUPS

1.1 Project Office Operations Committee
Dominik Mertz, Richard Whitlock, Jessica Vincent, Shun Fu Lee, Ingrid Copland

1.2 Steering Committee
Richard Whitlock (Chair), Alex Carignan, Stuart Connolly, PJ Devereaux, Mark Loeb, Dave Mazer, Mike McGillion, Dominik Mertz, Rohit Singal

1.3 Data and Safety Monitoring Board (DSMB)
Andrew Krahn (Chair), Charles Frenette, Jennie Johnstone, Jeff Healey, Melinda Musgrave

1.4 Adjudicators
Ali Alsagheir, Alex Carignan, Stephanie Smith

1.5 Project Office Staff
Sarah Apolcer, Ingrid Copland, Nikki Pinder, Jessica Vincent

1.6 Study Statisticians and Programmers
Shun Fu Lee, Peter Koh, Yueci Zhou

1.7 Participating Centres
CANADA (4,107) *Hamilton General Hospital (2007)*: Andre Lamy, Victor Chu, Adel Dyub, Iqbal Jaffer, Dominic Parry, Lloyd Semelhago, Richard Whitlock, Li Zhang, Heather Bergen, Patricia Power, Faye Browne; *London Health Sciences Centre (2100):* Mackenzie Quantz, Stephanie Fox, Carlee Stokes, Michael Chu, David Drullinsky, Martin Goldbach, Linrui Guo, Bob Kiaii, Dave Nagpal, Matthew Valdis
